# Extracellular vesicle digital scoring assay for assessment of treatment responses in hepatocellular carcinoma patients

**DOI:** 10.1186/s13046-025-03379-7

**Published:** 2025-05-01

**Authors:** Chen Zhao, Yi-Te Lee, Andrew Melehy, Minhyung Kim, Jacqueline Ziqian Yang, Ceng Zhang, Jina Kim, Ryan Y. Zhang, Junseok Lee, Hyoyong Kim, Yong Ju, Yuan-Jen Tsai, Xianghong Jasmine Zhou, Steven-Huy B. Han, Saeed Sadeghi, Richard S. Finn, Sammy Saab, David S. Lu, Jason Chiang, Jae-Ho Park, Todd V. Brennan, Steven A. Wisel, Manaf Alsudaney, Alexander Kuo, Walid S. Ayoub, Hyunseok Kim, Hirsh D. Trivedi, Yun Wang, Aarshi Vipani, Irene K. Kim, Tsuyoshi Todo, Justin A. Steggerda, Georgios Voidonikolas, Kambiz Kosari, Nicholas N. Nissen, Rola Saouaf, Amit G. Singal, Myung Shin Sim, David A. Elashoff, Sungyong You, Vatche G. Agopian, Ju Dong Yang, Hsian-Rong Tseng, Yazhen Zhu

**Affiliations:** 1 Department of Pathology and Laboratory Medicine, David Geffen School of Medicine, University of California, Los Angeles (UCLA), Los Angeles, CA 90095 USA; 2 Department of Molecular and Medical Pharmacology, California Nanosystems Institute, Crump Institute for Molecular Imaging, University of California, Los Angeles (UCLA), Los Angeles, CA 90095 USA; 3 Cancer Center, Renmin Hospital of Wuhan University, Wuhan, 430060 People’s Republic of China; 4 Karsh Division of Gastroenterology and Hepatology, Cedars-Sinai Medical Center, Los Angeles, CA 90048 USA; 5 Department of Surgery, David Geffen School of Medicine, University of California, Los Angeles (UCLA), Los Angeles, CA 90095 USA; 6 Department of Urology and Computational Biomedicine, Cedars-Sinai Medical Center, Los Angeles, CA 90048 USA; 7 Department of Family Medicine, Taipei Medical University Hospital, Taipei, 110301 Taiwan; 8 Department of Medicine, David Geffen School of Medicine, University of California, Los Angles (UCLA), Los Angeles, CA 90095 USA; 9 Department of Interventional Radiology, David Geffen School of Medicine, University of California, Los Angeles (UCLA), Los Angeles, CA 90095 USA; 10 Department of Surgery, Cedars-Sinai Medical Center, Los Angeles, CA 90048 USA; 11 Comprehensive Transplant Center, Cedars-Sinai Medical Center, Los Angeles, CA 90048 USA; 12 Department of Radiology, Cedars-Sinai Medical Center, Los Angeles, CA 90048 USA; 13 Division of Digestive and Liver Diseases, Department of Internal Medicine, University of Texas Southwestern Medical Center, Dallas, TX 75390 USA; 14 Department of Medicine Statistics Core, David Geffen School of Medicine, University of California, Los Angeles (UCLA), Los Angeles, CA 90095 USA; 15 Cedars-Sinai Medical Center, Samuel Oschin Comprehensive Cancer Institute, Los Angeles, CA 90048 USA; 16 Jonsson Comprehensive Cancer Center, University of California, Los Angeles (UCLA), Los Angeles, CA 90095 USA

**Keywords:** Hepatocellular Carcinoma, Extracellular Vesicle, Treatment Responses, Liquid Biopsy

## Abstract

**Background:**

There are no validated biomarkers for assessing hepatocellular carcinoma (HCC) treatment response (TR). Extracellular vesicles (EVs) are promising circulating biomarkers that may detect minimal residual disease in patients with treated HCC.

**Methods:**

We developed the HCC EV TR Score using HCC EV Digital Scoring Assay involving click chemistry-mediated enrichment of HCC EVs, followed by absolute quantification of HCC EV-specific genes by RT-digital PCR. Six HCC EV-specific genes were selected and validated through i) a comprehensive data analysis pipeline with an unprecedentedly large collection of liver transcriptome datasets (*n* = 9,160), ii) RNAscope validation on HCC tissues (*n* = 6), and iii) a pilot study on early- or intermediate-stage HCC and liver cirrhosis patients (*n* = 70). The performance of HCC EV TR Score was assessed in a phase-2 retrospective case–control study (n = 100).

**Results:**

HCC EV TR Scores, calculated from pre- and post-treatment plasma samples in the phase-2 case–control study, accurately differentiated post-treatment viable from nonviable HCC in the training (area under the ROC curve [AUROC] of 0.90, n = 49) and validation set (AUROC of 0.88, n = 51). At an optimal cutoff of 0.76 identified in the training set, HCC EV TR Score had high accuracy in detecting viable tumors (sensitivity: 76.5%, specificity: 88.2%) and found residual disease not initially observed on MRI in six patients with a median lead time of 63 days.

**Conclusions:**

This EV-based digital scoring approach shows great promise to augment cross-sectional imaging for the assessment of HCC treatment response.

**Supplementary Information:**

The online version contains supplementary material available at 10.1186/s13046-025-03379-7.

## Background

Despite recent advancements in treatment, the long-term prognosis of patients with hepatocellular carcinoma (HCC) remains poor [1–3]. For patients with early-stage HCC, surgical resection, liver transplantation (LT), and local ablation therapies are considered curative therapies. For intermediate-stage HCC, locoregional therapies, including transarterial chemoembolization (TACE) and transarterial radioembolization (TARE), are minimally invasive strategies that can prolong overall survival while preserving liver function [4]. Following these treatments, cross-sectional imaging, including computed tomography (CT) and magnetic resonance imaging (MRI), are employed to assess treatment response (TR) for determining prognosis and guiding further management [5]. Unlike surgical resection and LT, post-treatment (Tx) changes in the background liver parenchyma following locoregional therapies can complicate HCC TR assessment because local tumor damage from thermal, ischemic or radiation injury can lead to arterial phase hyperenhancement on cross-sectional imaging, which may take months to dissipate [6]. Currently, imaging-based criteria, including the modified Response Evaluation Criteria in Solid Tumors (mRECIST) [7], European Association for the Study of the Liver (EASL) criteria [8], and Liver Imaging Reporting and Data System Treatment Response (LR-TR) algorithm, are used to determine HCC TR assessment [9, 10]. However, assessment of response and residual tumor burden on cross-sectional imaging is often discordant with histologic tumor necrosis [11]. Additionally, persistent post-Tx arterial phase hyperenhancement [12] (i.e., equivocal response per LI-RADS) leads to more frequent follow-up imaging, which is associated with psychological distress as well as financial burden to patients and health systems [13].

Alpha-fetoprotein (AFP), the most widely studied serum biomarker for HCC, correlates with viable tumor burden [14]; however, only half of patients with HCC have elevated AFP prior to Tx[15], limiting its utility to assess HCC TR. Extracellular vesicles (EVs) are phospholipid bilayer-enclosed vesicles secreted by tumor and other cell types [16]. Tumor-derived EVs are detectable in peripheral blood during the nascent stages of malignancy, persisting through the continuum of disease progression. Quantification of tumor EVs [17] and their biomolecular cargoes, including nucleic acids [18] and proteins [19] could enable noninvasive detection of early-stage disease as well as dynamic TR assessment in solid tumors like HCC [20]. We recently demonstrated novel click chemistry-mediated EV enrichment technologies, i.e., EV Click Chips and EV Click Beads, enable downstream biomolecular characterizations of HCC-derived EVs in patient plasma. For example, the mRNA payloads in the enriched HCC EVs can be quantified [21] by reverse-transcription digital PCR (RT-dPCR). Given the capacity of the integrated HCC EV Digital Scoring Assay (i.e., EV Click Chips + RT-dPCR) [21] in accurately detecting early-stage HCC, we hypothesized that it could be adopted for TR assessment in HCC patients who received surgical resection, LT, or locoregional therapies (local ablation, TACE, and TARE).

**Fig. 1:**
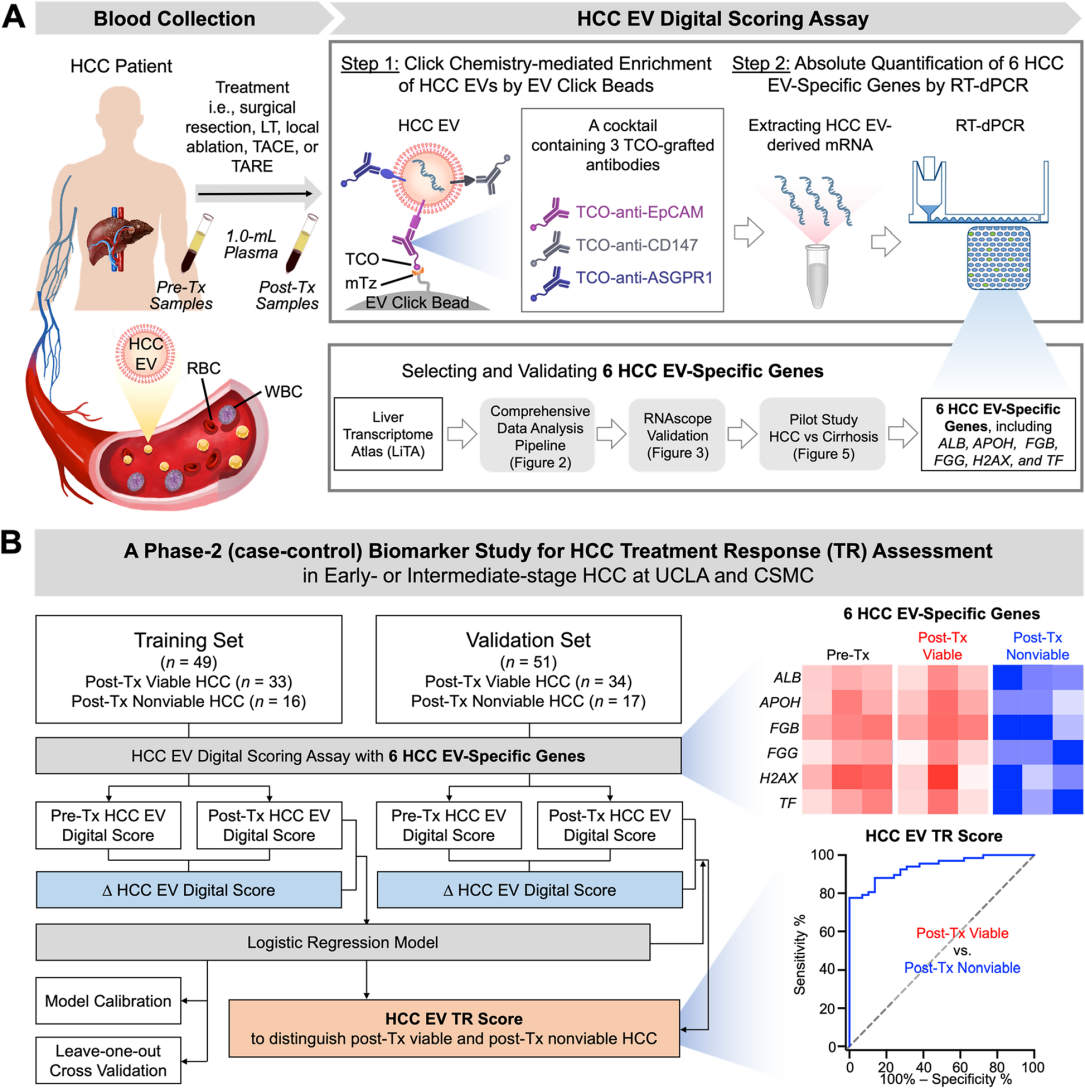
An HCC EV Digital Scoring Assay for HCC TR assessment. **A** The HCC EV Digital Scoring Assay is composed of a two-step workflow – Step 1: click chemistry-mediated enrichment of HCC EVs in 1.0-mL plasma by EV Click Beads in conjunction with the use of a cocktail of three TCO-grafted antibodies, and Step 2: absolute quantification of six HCC EV-specific genes by RT-dPCR. These six HCC EV-specific genes were selected and validated through i) a comprehensive data analysis pipeline based on our proprietary LiTA database, ii) RNAscope validation on HCC tissues and iii) a pilot study on early- or intermediate-stage HCC and liver cirrhosis patients. **B** A phase-2 retrospective case–control biomarker study was developed to evaluate the refined HCC EV Digital Scoring Assay for HCC TR assessment (i.e., distinguish post-Tx viable from nonviable HCC). Pre- and post-Tx plasma samples were collected from 100 patients with early- or intermediate-stage HCC, who received surgical resection, LT, local ablation, TARE, or TACE. The clinical status of these patients was classified as either post-Tx viable or nonviable in a 2:1 ratio. These patients were randomly divided into a training set (*n* = 49) and a validation set (*n* = 51). In the training set, all plasma samples were subjected to HCC EV Digital Scoring Assay to generate the respective pre-Tx and post-Tx HCC EV Digital Scores, and ∆ HCC EV Digital Scores (by subtracting the post-Tx with pre-Tx Scores). A logistic regression model was applied to integrate post-Tx HCC EV Digital Scores and ∆ HCC EV Digital Scores to establish HCC EV TR Scores, enabling effective differentiation of post-Tx viable and nonviable HCC. Subsequently, the study on the validation set reproduced the diagnostic efficacy of the HCC EV TR Scores for HCC TR assessment. ASGPR1, asialoglycoprotein receptor 1; CSMC, Cedars-Sinai Medical Center; EpCAM, epithelial cellular adhesion molecule; EV, extracellular vesicle; HCC, hepatocellular carcinoma; LiTA, Liver Transcriptome Atlas; LT, liver transplantation; mTz, methyltetrazine; RBC, red blood cell; RT-dPCR, reverse-transcription digital PCR; TACE, transarterial chemoembolization; TARE, transarterial radioembolization; TCO, trans-cyclooctene; TR, treatment response; Tx, treatment; UCLA, University of California, Los Angeles; WBC, white blood cell

Based on our previous work demonstrating the feasibility of using HCC EV-derived molecular signature for early detection [21, 22], we refined and validated the HCC EV Digital Scoring Assay using EV Click Beads and RT-dPCR for TR assessment (Fig. 1A) in HCC patients who received surgical resection, LT, or locoregional therapies (i.e., local ablation, TACE, or TARE). This assay was performed through a two-step workflow—Step 1: click chemistry-mediated enrichment of HCC EVs in 1.0-mL plasma by methyltetrazine (mTz)-grafted EV Click Beads [22] in conjunction with the use of a 3 trans-cyclooctene (TCO)-grafted antibody cocktail targeting three HCC EV surface markers (EpCAM, CD147, and ASGPR1), and Step 2: Absolute quantification of six HCC EV-specific genes (*ALB*, *APOH*, *FGB*, *FGG*, *H2AX*, and *TF*) by RT-dPCR. These six HCC EV-specific genes were selected and validated through i) a comprehensive data analysis pipeline based on our proprietary Liver Transcriptome Atlas (LiTA) database, which is unprecedently large collection of liver transcriptome datasets, and publicly available datasets, ii) RNAscope validation on HCC tissues, and iii) a pilot study on HCC (early- or intermediate-stage) and liver cirrhosis patients, where the disease window of the HCC patients matched that of the TR assessment HCC cohort. Subsequently, a phase-2 retrospective case–control biomarker study was developed (Fig. 1B) to evaluate the performance of the refined HCC EV Digital Scoring Assay for HCC TR assessment (i.e., distinguishing post-Tx viable from post-Tx nonviable HCC). Pre- and post-Tx plasma samples were collected from 100 early- or intermediate-stage HCC patients, which fell in either post-Tx viable or nonviable in a ratio of 2:1. These patients were then randomly divided into a training set (*n* = 49) and a validation set (*n* = 51). In the training set, all plasma samples were subjected to HCC EV Digital Scoring Assay to generate the respective pre-Tx and post-Tx HCC EV Digital Scores, and ∆ HCC EV Digital Scores (by subtracting the post-Tx with pre-Tx Scores). Finally, a logistic regression model was employed to combine the post-Tx HCC EV Digital Scores and ∆ HCC EV Digital Scores to establish HCC EV TR Scores, enabling effective differentiation of post-Tx viable and nonviable HCC. Subsequently, we validated the performance of HCC EV TR Scores on the validation set, confirming the diagnostic accuracy for HCC TR assessment.

## Materials and Methods

### Development of the LiTA database

**Fig. 2:**
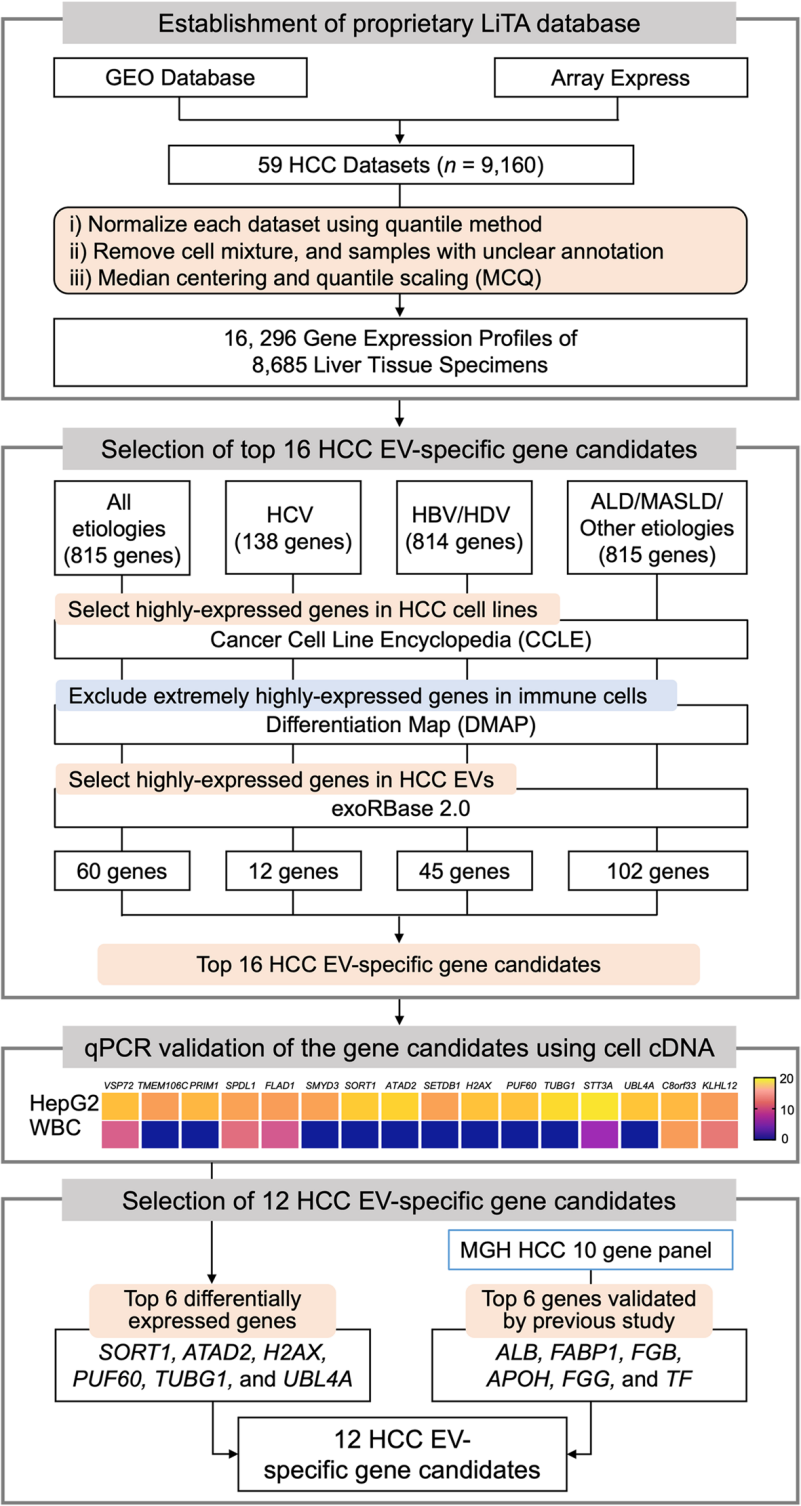
A comprehensive data analysis pipeline for identifying 12 HCC EV-specific gene candidates. The pipeline begins with the preparation of our proprietary LiTA database, which was established by aggregating publicly available liver transcriptome data from 59 cohorts (*n* = 9,160) obtained from the GEO and Array Express repositories, resulting in 16,296 gene expression profiles for 8,685 liver tissue specimens. In the gene selection process, the top 16 HCC EV-specific gene candidates were selected through the following four steps: i) selecting upregulated genes across four etiology-based comparisons; ii) selecting highly expressed genes in HCC cell lines using CCLE dataset; iii) excluding highly expressed genes in immune cells using DMAP dataset; iv) selecting highly expressed genes in HCC EVs using exoRBase 2.0. qPCR was employed to measure the differential expression of the top 16 HCC EV-specific gene candidates in HepG2 cells and WBCs (Scale: 40 – cycle threshold value). Six genes (*SORT1*, *ATAD2*, *H2AX*, *PUF60*, *TUBG1*, and *UBL4 A*) with the top-ranking differential expressions (high in HepG2 cells and low in WBCs) were selected. In parallel, another six genes (*ALB*, *FABP1*, *FGB*, *APOH*, *FGG*, and *TF*) with performance in distinguishing early-stage HCC from liver cirrhosis were also identified. Combining these findings, we identified the 12 HCC EV-specific gene candidates. ALD, alcoholic liver disease; CCLE, Cancer Cell Line Encyclopedia dataset; DMAP, Differentiation MAP dataset; EV, extracellular vesicle; GEO, Gene Expression Omnibus; HBV, hepatitis B virus; HCC, hepatocellular carcinoma; HCV, hepatitis C virus; HDV, hepatitis D virus; LiTA, Liver Transcriptome Atlas; MASLD, metabolic dysfunction-associated steatotic liver disease; MGH, Massachusetts General Hospital; qPCR, quantitative PCR; WBC, white blood cell

We collected 59 cohorts (*n* = 9,160) of liver transcriptome data from Gene Expression Omnibus (GEO) [23] and ArrayExpress [24], and assemble a merged dataset as a virtual cohort of liver transcriptome data, LiTA (Fig. 2). To this end, we applied the median-centering and quantile scaling (MCQ) method [25] to remove systemic, dataset-specific bias. Briefly, each dataset was first normalized using the quantile method [26]. The cell mixture and samples with unclear annotations were then removed from the dataset. Probes or transcripts were assigned to unique genes by mapping National Center for Biotechnology Information (NCBI) entrez gene IDs. Redundant replications for each probe and transcript were removed by selecting the one with the highest mean expression. Log_2_ intensities for each gene were centered by the median of all samples in the dataset. Each of the matrices was then transformed into a single vector. The vectors for the matrices were scaled by the quantile method to avoid a bias toward certain datasets or batches with large variations from the median values. These scaled vectors were transformed back into the matrices. The matrices were combined by matching the gene IDs in the individual matrices.

## RNAscope ISH

To localize mRNA of 12 HCC EV-specific gene candidates, we employed the RNA–Protein Co-Detection Ancillary kit (Cat. No. 323180, Advanced Cell Diagnostics [ACD] RNAScope™) in combination with immunofluorescence on formalin-fixed, paraffin-embedded (FFPE) tissue sections of HCC tissues compared to nontumor margins. Following the manufacturer's instructions, paraffin-embedded slides were baked at 60 °C for 1 h and subsequently deparaffinized in xylene and 100% ethanol. Endogenous peroxidase activity was blocked with H_2_O_2_ for 10 min at room temperature. Antigen retrieval was performed using RNAscope Target Retrieval Reagents for 15 min in a steam cooker. The slides were then transferred to fresh distilled water, washed with Phosphate Buffered Saline with Tween 20 (PBS-T), and incubated with the anti-CD147 primary antibody overnight at 4 °C. Following incubation, the slides were washed with PBS-T, fixed in 4% paraformaldehyde for 30 min at room temperature, and treated with RNAscope Protease Plus for 30 min at 40 °C in an ACD Hybridizer. After washing with RNAscope Wash Buffer Reagents, the slides were incubated at 40 °C for 2 h with the respective 12 primary probes. Following extensive washing, the slides were subjected to amplification probes, with detection using Opal™ fluorophores. A fluorophore-conjugated secondary antibody diluted in Co-Detection Antibody Diluent was applied to completely cover the sections. Finally, 4′,6-diamidino- 2-phenylindole (DAPI) was added to each slide, followed by 1–2 drops of Prolong Gold antifade mounting medium. Detailed procedures for image acquisition and analysis are provided in **Supplementary Methods**.

## General information for HCC EV Digital Scoring Assay

The HCC EV Digital Scoring Assay is implemented through a two-step workflow (Fig. 1) as described below:

Step 1: Click chemistry-mediated enrichment of HCC EVs by EV Click Beads. TCO-antibody conjugates were prepared as in our previous study [21]. A 1.0-mL clinical or synthetic plasma sample was incubated with the cocktail (14 µL) of three TCO-grafted HCC EV-associated antibodies (TCO-anti-EpCAM [200 ng], TCO-anti-CD147 [250 ng], and TCO-anti-ASGPR1 [500 ng]) at room temperature for 45 min. The plasma sample was then incubated with the EV Click Beads [22] (0.1 mg) for 45 min for enrichment of HCC EVs. The EV Click Beads with enriched EVs were collected by centrifuge at 10,000 g for 2 min.

Step 2: Absolute quantification of HCC EV-specific genes by RT-dPCR. The collected EV Click Beads with enriched EVs were lysed using 700 µL of QIAzol Lysis Reagent. RNA was extracted with a miRNeasy Micro Kit (Qiagen, USA). cDNA synthesis was performed using a Thermo Scientific Maxima H Minus Reverse Transcriptase Kit. To confirm the specificity of HCC EV-associated antibody for click chemistry-mediated enrichment of HCC EVs, we adopted a quantitative RT-dPCR method [21] in conjunction with the use of synthetic plasma samples prepared by spiking HepG2 EVs into the plasma from a female healthy donor (HD). The results shown in Supplementary Fig. S1 suggest that HCC EV-associated antibody was able to enrich HepG2 EVs with high purity, validating the capture specificity of HCC EV-associated antibody for click chemistry-mediated enrichment of HCC EVs. To ensure the feasibility of triplex dPCR assay (QIAcuity Digital PCR System, Qiagen, USA), PCR primers and probes were validated using cDNA obtained from HepG2 cells, which was tested for 12 HCC EV-specific gene candidates using triplex qPCR (see details in Supplementary Fig. S2). In the pilot study, the expression levels of 12 HCC EV-specific gene candidates were evaluated. For the phase-2 biomarker study, the analysis focused on the 6 HCC EV-specific genes that demonstrated differential expression in the pilot study.

## Study design and patient cohorts

To select top-ranking HCC EV-specific genes from the 12 HCC EV-specific gene candidates, we carried out a pilot study to assess and optimize the performance of the HCC EV Digital Scoring Assay in distinguishing early- or intermediate-stage. A phase-2 biomarker study was designed to establish and validate HCC EV TR Scores for differentiating post-Tx viable from nonviable HCC following surgical or locoregional therapies. Pre- and post-Tx plasma samples from each HCC patient were collected from both Ronald Reagan University of California, Los Angeles (UCLA) Medical Center and Cedars-Sinai Medical Center (CSMC) and were subjected to the optimized HCC EV Digital Scoring Assay. All clinical plasma experiments were performed in a blinded manner to eliminate bias in the assessment. No outliers or other data points were excluded from the analysis. Before processing clinical samples, a comprehensive data analysis pipeline was employed to identify the 12 HCC EV-specific gene candidates. This was followed by RNAscope in situ hybridization and immunofluorescence validation. Furthermore, extensive analysis of HCC cell-derived EVs and optimization studies were carried out to validate and refine the HCC EV Digital Scoring Assay.

In the pilot study, 35 patients with Tx-naïve Barcelona Clinical Liver Cancer (BCLC) stage 0-B HCC and 35 patients with liver cirrhosis were enrolled between July 2019 and February 2022 at Ronald Reagan UCLA Medical Center. For the phase-2 biomarker study, 100 patients with early- or intermediate-stage HCC receiving Tx including surgical resection, LT, local ablation, TARE, or TACE, were enrolled between August 2019 and December 2023 at Ronald Reagan UCLA Medical Center and CSMC. Using R ‘sample’ function, these patients were randomly divided into a training set (*n* = 49, including 33 post-Tx viable and 16 post-Tx nonviable) and a validation set (*n* = 51, including 34 post-Tx viable and 17 post-Tx nonviable). Early-stage HCC was defined as BCLC stage 0 or A, while intermediate-stage HCC corresponded to BCLC stage B. The detailed description of inclusion criteria for the included cohorts can be found in the Supplementary Methods. All participants provided written informed consent for this study according to the institutional review board (IRB) protocols #14–000197, #10–000236-AM- 00021 and #20–001197 at UCLA, and IRB protocols #00000066, #00042197, and #00033050 at CSMC. Samples were collected only after obtaining written informed consent from the participants.

Post-Tx samples were collected a minimum of four weeks following Tx to mitigate any inflammatory effects on EV secretion due to the Tx. Patients were excluded from the study if they had concurrent neoplasms or a history of malignancy within the past five years. The post-Tx tumor status was classified as either "viable" or "nonviable" based on: i) cross-sectional imaging (CT/MRI) at the first post-Tx blood draw as determined by the LR-TR algorithm [27], ii) explant histology results for LT patients, or iii) early recurrence, defined as recurrence within 180 days post-Tx. For patients with equivocal responses according to the LR-TR algorithm, follow-up imaging was used to confirm tumor viability.

## Blood processing

Peripheral venous blood samples were collected from participants with written informed consent according to the IRB protocols at UCLA and CSMC. A 10.0-mL ethylenediaminetetraacetic acid vacutainer tube (BD Medical, Fisher Cat. #BD 366643–1) was used for blood sample collection. The whole blood was first centrifuged at 530 × g (4 °C) for 10 min to remove cells and cell debris. The supernatant was collected in new tubes and centrifuged at 4600 × g (4 °C) for 10 min to further eliminate the remaining cellular debris and large particles. The two centrifugation steps ensure that no cell particles and debris were left in the plasma samples. The final cell-free plasma supernatant was collected and stored at − 80 °C until further use for the HCC EV Digital Scoring Assay.

## Statistical analysis

For descriptive statistics, continuous variables are presented as medians and interquartile ranges, while categorical variables are reported as numbers and percentages. The Mann–Whitney U test and Fisher exact test or chi-square test were used to compare continuous and categorical variables between groups, respectively.

The sample size was calculated according to the area under receiver operating characteristic curve (AUROC) comparison between our assay and serum AFP using the paired DeLong’s test. A sample size of 48 (32 cases of viable and 16 cases of nonviable, the ratio of sample sizes in nonviable/viable groups is 1/2) is expected to have 80% power to detect the difference between the AUROCs for our assay versus serum AFP, assuming AUROC = 0.86 for our assay, AUROC = 0.63 for serum AFP [28], when a correlation between the assays of 0.5 was assumed. The power was obtained for a two-sided test at 0.05 significance level.

To determine the HCC EV TR Score for TR assessment, logistic regression analysis was applied in the training set, which synergistically combined the post-Tx HCC EV Digital Score and ∆ HCC EV Digital Score. Receiver operating characteristic (ROC) curve analysis evaluated the performance of the HCC EV Digital Score in the pilot study and the HCC EV TR Score in the training and validation sets. Youden's index was used to identify the optimal cutoff of HCC EV Digital Score and HCC EV TR Score, respectively. We also included relevant patient demographics and clinical factors as covariates in our statistical analyses. More statistical methods and data availability were described in the Supplementary Method.

All statistical analyses were performed using R statistical software (version 4.1.0; R Foundation, Vienna, Austria), GraphPad Prism (version 9.2.0; GraphPad Software, Inc., CA, USA), and IBM SPSS Statistics (IBM Corp., Armonk, NY, USA), with two-sided tests and a significance level of 0.05. The"integrated waterfall/swimmer plots"were created using Python 3.12.4.

## Results

### Identification of 12 HCC EV-specific gene candidates

We established a comprehensive data analysis pipeline to identify HCC EV-specific gene markers (Fig. 2). This pipeline begins with the unprecedently large collection of liver transcriptome datasets from the GEO and ArrayExpress repositories. This compilation resulted in 16,296 gene expression profiles from 8,685 liver tissue specimens, referred to as LiTA.

In the gene selection process, the following four steps were employed (Fig. 2, detailed in the Supplementary Methods): i) selecting of upregulated genes in HCC tissues across four etiology-based comparisons; ii) selecting of highly expressed genes in HCC cell lines using the Cancer Cell Line Encyclopedia (CCLE) dataset [29]; iii) excluding of highly expressed genes in immune cells through the Differentiation MAP (DMAP) dataset [30]; and iv) selecting of highly expressed genes in HCC EVs using HCC EV RNA sequencing data from exoRBase 2.0 [31]. This multi-step approach yielded top 16 HCC EV-specific gene candidates (*VPS72*, *TMEM106C*, *PRIM1*, *SPDL1*, *FLAD1*, *SMYD3*, *SORT1*, *ATAD2*, *SETDB1*, *H2AX*, *PUF60*, *TUBG1*, *STT3A*, *UBL4A*, *C8orf33*, and *KLHL12*).

We then performed quantitative PCR (qPCR) to measure the differential expression of the selected top 16 HCC EV-specific gene candidates in a human HCC cell line (HepG2) and in white blood cells (WBCs) from an HD. Six genes (*SORT1*, *ATAD2*, *H2AX*, *PUF60*, *TUBG1*, and *UBL4A*) that exhibited top-ranking differential expressions were selected, with high expression in HepG2 cells and low expression in WBCs (see heatmap in Fig. 2). Additionally, another six genes (*ALB*, *APOH*, *FABP1*, *FGB*, *FGG*, and *TF*) were identified from our previous HCC EV-specific gene panel [21] given their performance in distinguishing early-stage HCC from liver cirrhosis. This panel was originally adapted from Massachusetts General Hospital’s digital detection platform [32] for HCC circulating tumor cells. The resultant combined 12 HCC EV-specific gene candidates are: *ALB*, *APOH*, *ATAD2*, *FABP1*, *FGG*, *FGB*, *H2AX*, *PUF60*, *SORT1*, *TF*, *TUBG1*, and *UBL4A*. This comprehensive data analysis pipeline ensures that the selected genes are specific to HCC EVs and can effectively differentiate themselves from those in the blood background.

## Validation of the 12 HCC EV-specific gene candidates in HCC tissues using RNAscope

**Fig. 3:**
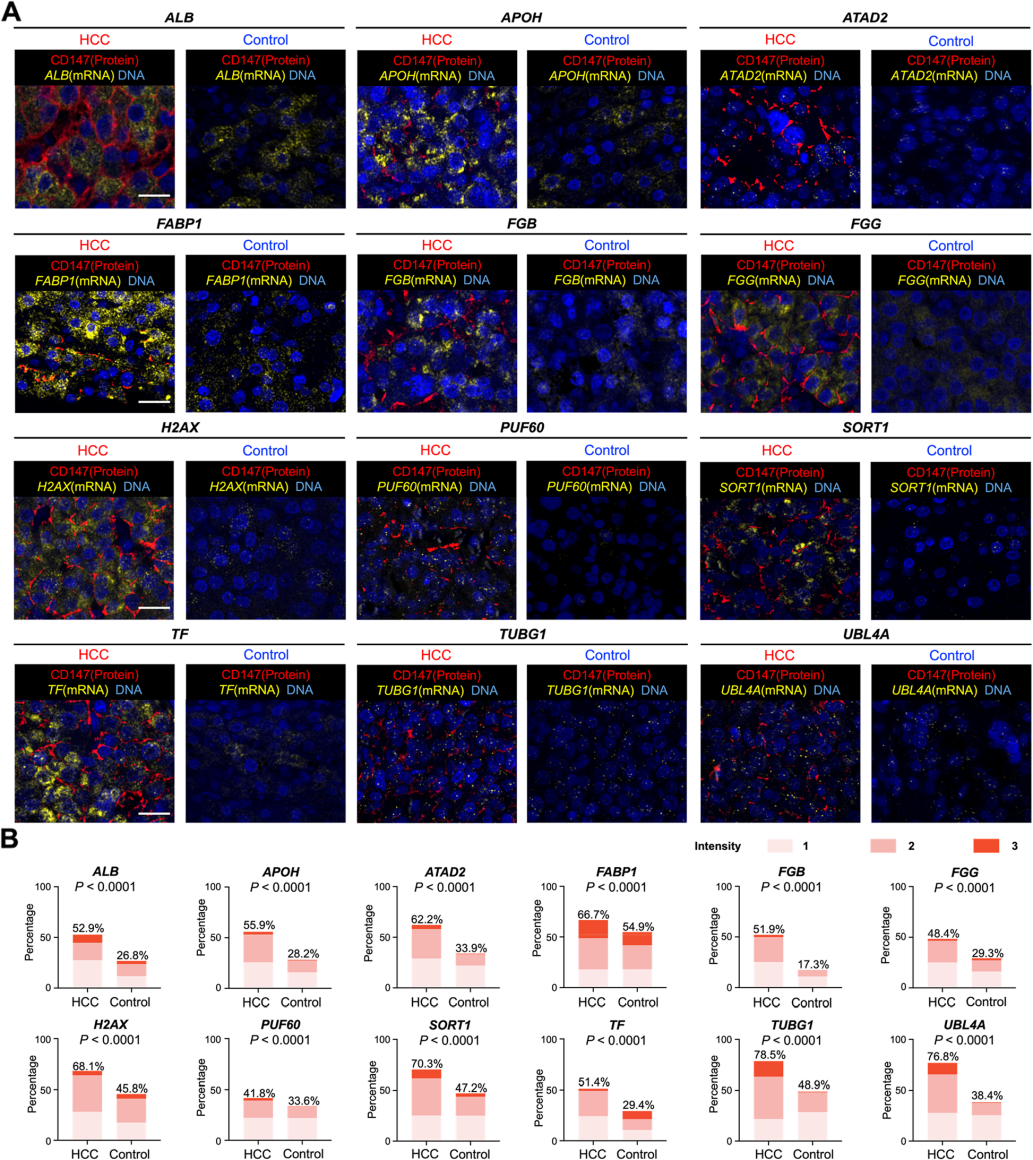
Validation of 12 HCC EV-specific gene candidates using RNAscope. **A** Representative RNAscope in situ hybridization for 12 HCC EV-specific mRNA candidates and immunofluorescent staining of CD147 (HCC-associated surface protein marker) were performed on HCC tissues and their corresponding negative surgical margins. **B** The percentage of cells with positive mRNA staining (intensity levels 1 to 3) for the 12 markers is summarized in a bar chart. Statistical significance between groups was determined using the Chi-square test. EV, extracellular vesicle; HCC, hepatocellular carcinoma

To further validate whether the 12 HCC EV-specific gene candidates are differentially expressed within HCC tissues compared to nontumor margins, we employed RNA–Protein Co-Detection Ancillary kit that allows for simultaneous RNAscope in situ hybridization (ISH) and immunofluorescence. Six FFPE HCC tissues and their respective negative surgical margins were acquired for this study. Specifically, the custom-ordered RNAscope ISH probes are capable of detecting and quantifying each of the 12 HCC EV-specific gene candidates in these tissues. In parallel, immunofluorescence of CD147 can delineate the HCC cell membrane to confirm the presence of HCC cells. With superb spatial resolution, the resultant merged fluorescence micrographs (Fig. 3A) allowed for semi-quantitative analysis of differential expression levels (Fig. 3B) among the 12 gene candidates between HCC tissues and their nontumor margins. These results further confirmed the differential expression of the 12 HCC EV-specific gene candidates within HCC tissues and laid a solid foundation for the use of these genes for developing the HCC EV Digital Scoring Assay.

## Linearity studies of HCC EV Digital Scoring Assay for quantifying each of the 12 HCC EV-specific gene candidates

**Fig. 4:**
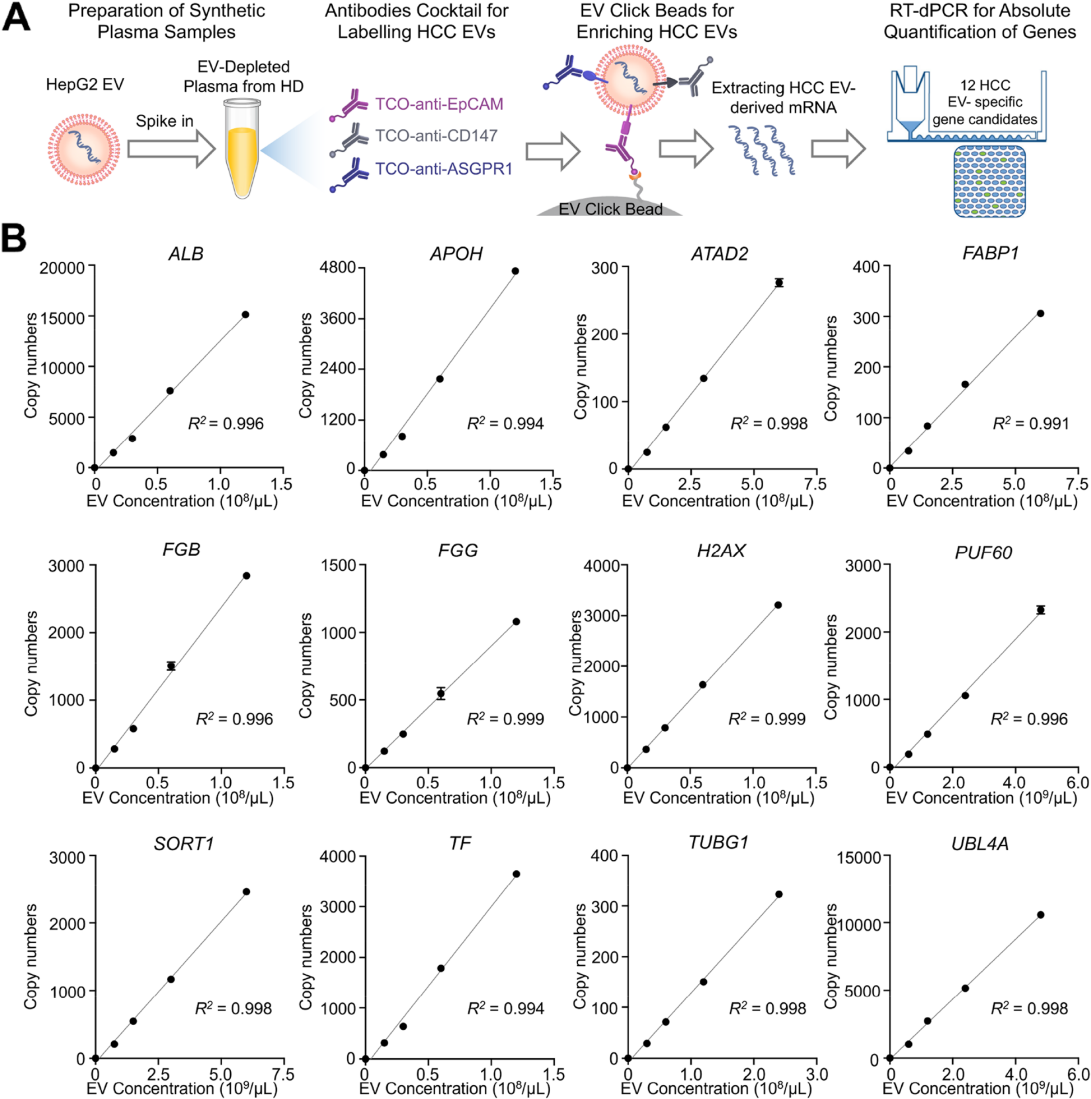
Linearity studies of HCC EV Digital Scoring Assay. **A** Linearity studies of HCC EV Digital Scoring Assay using synthetic plasma sample, prepared by serially spiking HepG2 EVs into the EV-depleted HD plasma. EV Click Beads were utilized to enrich HepG2 EVs in conjunction with the cocktail of the 3 TCO-antibodies targeting EpCAM, CD147, and ASGPR1. RT-dPCR was performed to obtain the absolute quantification of 12 HCC EV-specific gene candidates. **B** Robust linearity correlations were identified between the concentration of spiked HepG2 EVs and measured mRNA copy numbers for all 12 HCC EV-specific gene candidates over the concentration range of 0 – 6 × 10^9^ HepG2 EVs per µL, with R-squared values ranging from 0.991 to 0.999. ASGPR1, asialoglycoprotein receptor 1; EpCAM, epithelial cellular adhesion molecule; EV, extracellular vesicle; HCC, hepatocellular carcinoma; HD, healthy donor; RT-dPCR, reverse-transcription digital PCR; TCO, trans-cyclooctene

As a model system, HCC cell-derived EVs were harvested from the conditioned media of HepG2 cells using ultracentrifugation [21]. The characterization of the resultant HepG2 EVs (Supplementary Fig. S3) adhered to the guidelines outlined by the International Society for Extracellular Vesicles minimal information for studies of extracellular vesicles (MISEV 2018) [33]. HepG2 EVs were characterized using transmission electron microscopy (TEM) and nanoparticle tracking analysis (NTA). These findings collectively validated that EV Click Beads can be employed for click chemistry-mediated immobilization of HCC EVs. We then proceeded to validate the two-step HCC EV Digital Scoring Assay using synthetic plasma samples. As illustrated in Fig. 4A, synthetic plasma samples were prepared by serially spiking 10 µL of HepG2 EVs into 90 µL of EV-depleted female HD plasma. The quantities of HepG2 EVs in the stock solution were determined by measuring the EV counts using NTA. In the 0.1-mL synthetic plasma, the spiked HepG2 EVs were incubated and labeled with a 14-µL antibody cocktail containing TCO-anti-EpCAM (200 ng), TCO-anti-CD147 (250 ng), and TCO-anti-ASGPR1 (500 ng), followed by enrichment using EV Click Beads. The enriched HepG2 EVs were then subjected to triplex RT-dPCR for the absolute quantification of each of the 12 HCC EV-specific gene candidates, including *ALB*, *APOH*, *ATAD2*, *FABP1*, *FGB*, *FGG*, *H2AX*, *PUF60*, *SORT1*, *TF*, *TUBG1*, and *UBL4A*. As shown in Fig. 4B, robust linear correlations (R^2^ > 0.90) were observed between the levels of spiked HepG2 EVs and the detected mRNA copy numbers across all 12 HCC EV-specific gene candidates over the concentration range of 0 – 6 × 10^9^ HepG2 EVs per µL, with R-squared values ranging from 0.991 to 0.999.

## A pilot study to select the 6 HCC EV-specific genes from the 12 HCC EV-specific gene candidates and calculate HCC EV Digital Scores

**Fig. 5:**
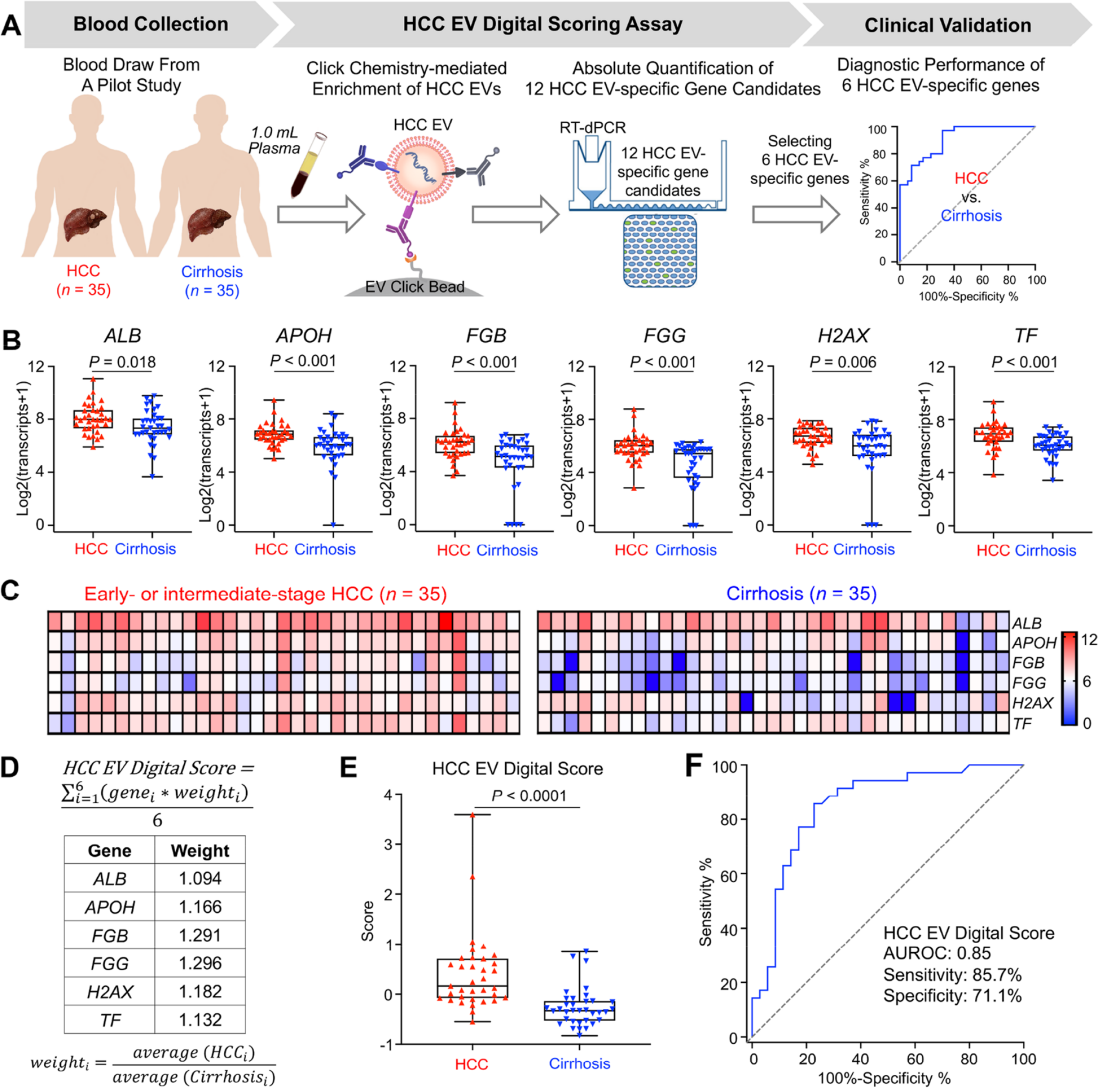
A pilot study to refine the 6 HCC EV-specific genes from the 12 HCC EV-specific gene candidates and calculate HCC EV Digital Score. **A** To refine the 6 HCC EV-specific genes from the 12 HCC EV-specific gene candidates, a pilot study of HCC EV Digital Scoring Assay was conducted to differentiate early- or intermediate-stage Tx-naïve HCC (BCLC 0-B, *n* = 35) from liver cirrhosis (*n* = 35), where the disease window of the HCC cases matched that of the HCC TR assessment cohort. **B** The 6 differentially expressed genes *ALB*, *APOH*, *FGB*, *FGG*, *H2 AX*, and *TF*, capable of better differentiating early- or intermediate-stage HCC from liver cirrhosis were identified. **C** Heatmaps summarizing relative intensities of the 6 HCC EV-specific genes across the early- or intermediate-stage HCC and liver cirrhosis patients. Scale: log_2_(transcript + 1). **D** A weighted Z-score formula and the respective weights of the six genes were developed to integrate the 6-gene readouts into HCC EV Digital Scores. In this formula, *i* represents each gene, with weights assigned based on the relative signal-to-noise ratio in the average gene copies (log_2_ transformed) of HCC patients relative to those of cirrhosis patients. **E** Boxplot summarizing the HCC EV Digital Scores calculated for the early- or intermediate-stage HCC and liver cirrhosis patients. **F** Receiver operating characteristic (ROC) curves of HCC EV Digital Score for differentiating early- or intermediate-stage HCC and liver cirrhosis patients (AUROC = 0.85, 95% CI: 0.76—0.94). AUROC, area under the receiver operating characteristic curve; EV, extracellular vesicle; HCC, hepatocellular carcinoma; ROC, receiver operating characteristic; RT-dPCR, reverse-transcription digital PCR

To select top-ranking HCC EV-specific genes from the 12 HCC EV-specific gene candidates, we carried out a pilot study (Fig. 5A) where the performance of HCC EV Digital Scoring Assay for differentiating early- or intermediate-stage HCC from liver cirrhosis was evaluated and optimized. We collected 70 plasma samples from 35 patients with Tx-naïve early- or intermediate-stage HCC (BCLC 0-B) and 35 patients with cirrhosis controls without HCC, where the disease window of the HCC patients matched that of the HCC TR assessment cohort. The inclusion of early- or intermediate-stage HCC cases ensures that the selected top-ranking HCC EV-specific genes are detectable in HCC patients with low disease burden. In addition, to enhance the specificity of the selected top-ranking genes for detecting HCC, we enrolled liver cirrhosis patients as the controls since most HCC patients have an intrinsic background of cirrhosis. Demographic and clinical characteristics of the patients are described in Supplementary Table S1 and Table S2. For each sample, the enriched HCC EVs were then subjected to RT-dPCR for absolute quantification of each of the 12 HCC EV-specific gene candidates. We then analyzed the expressions of gene transcripts between HCC and liver cirrhosis (Fig. 5B, Supplementary Fig. S4). Among the 12 HCC EV-specific gene candidates, 6 genes were differentially expressed (*P* < 0.05, Fig. 5B), which were identified as *ALB*, *APOH*, *FGB*, *FGG*, *H2AX*, and *TF*. We summarized the relative intensities of these 6 HCC EV-specific genes across the 70 patients in heatmaps (Fig. 5C). Using a weighted Z-score method [21, 34], a formula and the respective weight of each gene [35] were generated (Fig. 5D) to integrate the 6-gene readouts into an HCC EV Digital Score for each patient. The weight of each gene was assigned based on the relative signal-to-noise ratio in the average gene copies (log_2_ transformed) of HCC patients relative to those of cirrhosis patients. The resultant HCC EV Digital Scores accurately differentiated early- or intermediate-stage HCC cases from controls with cirrhosis (*P* < 0.0001; Fig. 5E). Moreover, compared to the result observed for serum AFP (Supplementary Fig. S5), the HCC EV Digital Scores demonstrated improved performance in detecting early- or intermediate-stage HCC (AUROC = 0.85, 95% confidence interval [CI] = 0.76–0.94, sensitivity = 85.7%, specificity = 77.1%, Fig. 5F). Taken together, we successfully validated the HCC EV Digital Scoring Assay in the pilot study and generated an HCC EV Digital Score to quantify systemic HCC burden, paving a way for non-invasive HCC TR assessment.

## Establishment of HCC EV TR Scores for differentiating post-Tx viable and post-Tx nonviable HCC after surgical or locoregional therapies

We next evaluated the performance of the HCC EV Digital Score formula (Fig. 5D) for HCC TR assessment by distinguishing post-Tx viable from post-Tx nonviable HCC. A pair of pre-Tx and post-Tx plasma samples were collected from early- or intermediate-stage HCC patients who received surgical resection, LT, or locoregional therapies (i.e., local ablation, TACE, or TARE). Post-Tx disease status was determined using post-LT histology or LR-TR algorithm, classifying patients as either post-Tx viable or post-Tx nonviable. The characteristics of these 100 HCC patients are summarized in Table 1. The demographic and clinical characteristics, including age, sex, and race/ethnicity, BCLC stage, and therapies, were similar between the training and validation sets.

**Table 1:** Demographic and clinical characteristics of 100 early- or intermediate-stage HCC patients who received surgical resection, LT, or locoregional therapies

**Characteristics**	**Training Set** *n* = 49	**Validation Set** *n* = 51	***P* value**
**Age, median (IQR)**	67(59–74)	66(62–70.5)	0.95
**Male, n (%)**	33(67.3%)	33(64.7%)	0.78
**Post-Tx status**			0.94
Viable	33(67.3%)	34(66.7%)	
Nonviable	16(32.7%)	17(33.3%)	
**Race/ethnicity, n (%)**			0.27
Asian	11(22.4%)	7(13.7%)	
Black	5(10.2%)	1(2.0%)	
White	12(24.5%)	16(31.4%)	
Hispanic	20(40.8%)	23(45.1%)	
Others/Unknown	1(2.1%)	4(7.8%)	
**Cirrhosis, n (%)**	40 (81.6%)	47(92.2%)	0.12
**HCC etiology, n (%)**			0.04
HBV	7(14.2%)	2(3.9%)	
HCV	9(18.4%)	19(37.3%)	
ALD	9(18.4%)	7(13.7%)	
MASLD	15(30.6%)	16(31.4%)	
≥ 2 etiologies	0(0%)	3(5.9%)	
Others	9(18.4%)	4(7.8%)	
**BCLC stage, n (%)**			0.72
Stage 0	2(4.1%)	6(11.8%)	
Stage A	40(81.6%)	36(70.6%)	
Stage B	7(14.3%)	9(17.6%)	
**Tx option**			0.52
Resection	5(10.2%)	3(5.9%)	
LT	0(0%)	1(2.0%)	
Local ablation	14(28.5%)	17(33.3%)	
TACE	9(18.4%)	14(27.5%)	
TARE	21(42.9%)	17(33.3%)	
**Milan criteria, n (%)**			
Within Milan criteria	36 (73.5%)	43(84.3%)	0.18
Outside Milan criteria	13 (26.5%)	8(15.7%)	
**AFP, ng/mL, median (IQR)**	10.1 (3.4–91.8)	9.2(3.6–23.4)	0.26

*AFP* alpha-fetoprotein, *ALD* alcoholic liver disease, *BCLC* Barcelona clinic liver cancer, *HBV* Hepatitis B, *HCV* Hepatitis C, *IQR* interquartile range, *MASLD* metabolic dysfunction-associated steatotic liver disease, *LT* liver transplantation, *TACE* transarterial chemoembolization, *TARE* transarterial radioembolization

**Fig. 6:**
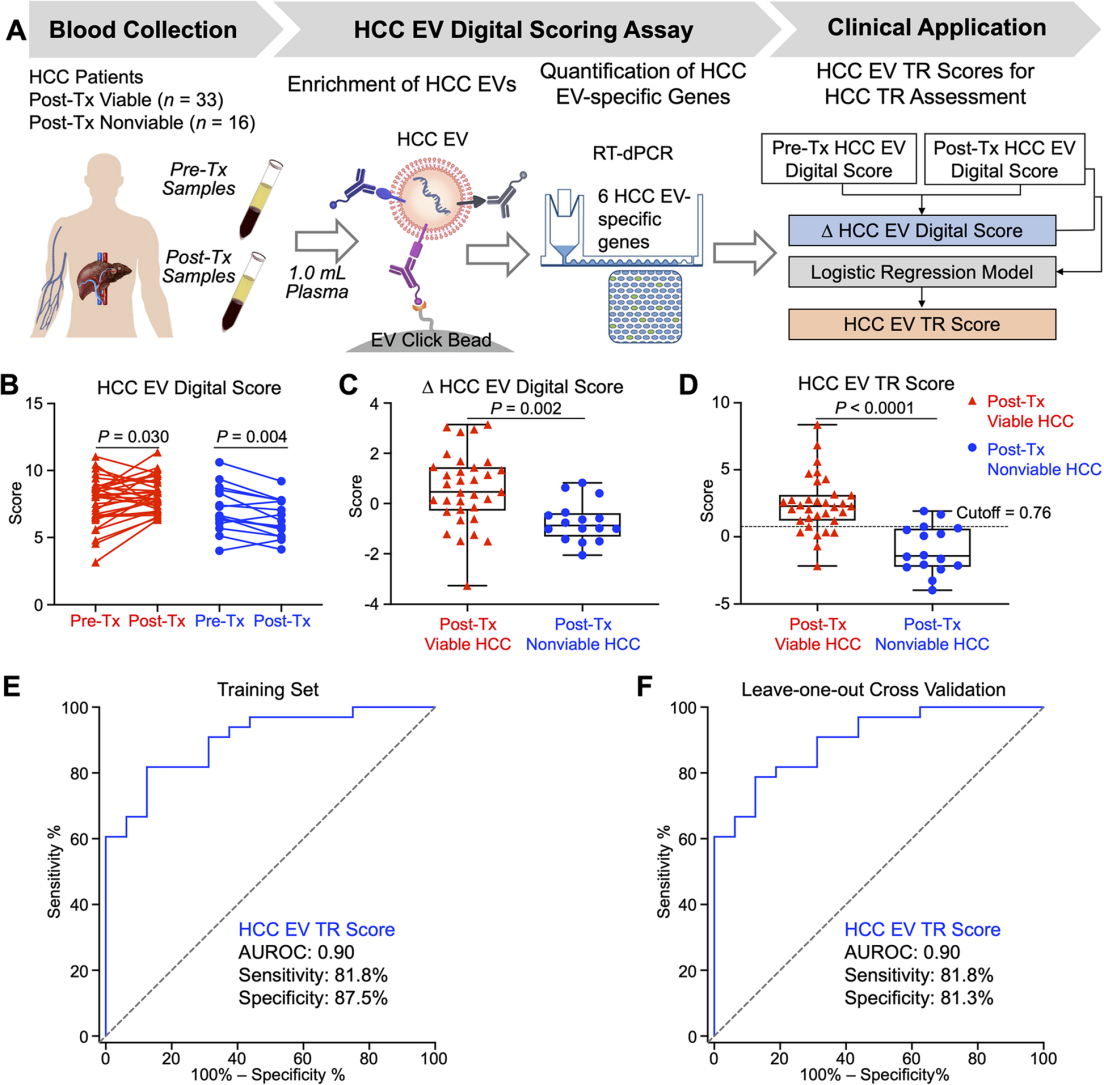
Establishment of HCC EV TR Scores for differentiating post-Tx viable and post-Tx nonviable HCC in the training set (*n* = 49). **A** Pre-Tx and post-Tx plasma samples from each HCC patient were subjected to the refined HCC EV Digital Scoring Assay to generate the respective pre-Tx and post-Tx HCC EV Digital Scores and calculate ∆ HCC EV Digital Scores by subtracting the post-Tx from pre-Tx Scores. A logistic regression model was developed to synergistically combine the post-Tx HCC EV Digital Scores and ∆ HCC EV Digital Scores to establish their HCC EV TR Scores. **B** Ladder plots summarizing the pre-Tx and post-Tx HCC EV Digital Scores for the 33 post-Tx viable HCC patients and 16 post-Tx nonviable HCC patients. **C/D** Boxplots summarizing ∆ HCC EV Digital Scores and HCC EV TR Scores of the 33 post-Tx viable HCC patients and the 16 post-Tx nonviable HCC patients. The dashed line indicates the optimal cutoff of 0.76. **E** ROC curve of HCC EV TR Score for differentiating post-Tx viable HCC from post-Tx nonviable HCC in the training set.** F** ROC curve after leave-one-out cross-validation for differentiating post-Tx viable HCC from post-Tx nonviable HCC in the training set. AUROC, area under receiver operating characteristic curve; EV, extracellular vesicle; HCC, hepatocellular carcinoma; LT, liver transplantation; ROC, receiver operating characteristic; RT-dPCR, reverse-transcription digital PCR; TACE, transarterial chemoembolization; TARE, transarterial radioembolization; TR, treatment response; Tx, treatment

In the training set (*n* = 49), pre-Tx and post-Tx plasma samples from each HCC patient were subjected to the refined HCC EV Digital Scoring Assay (Fig. 6A) to quantify 6 HCC EV-specific genes and generate paired pre-Tx and post-Tx HCC EV Digital Scores (Fig. 6B). Next, ∆ HCC EV Digital Score was calculated for each patient by subtracting their post-Tx from pre-Tx Score using the following equation:

$$HCC\ EV\ Digital\ Score=\frac{{\sum }_{i=1}^{6}\left(\left(gene_{post}-gene_{pre}\right)_{i}*weight_{i}\right)}{6}$$

where *i* represents each gene, with *weight* assigned based on the relative signal-to-noise ratio from the pilot study.

Post-Tx nonviable HCC showed significantly greater median decrease in ∆ HCC EV Digital Scores compared to post-Tx viable HCC (*P* = 0.002, Fig. 6C). A logistic regression model (see the formula below) was developed to synergistically combine the post-Tx HCC EV Digital Scores and the ∆ HCC EV Digital Scores to establish HCC EV TR Scores.

$$HCC\ EV\ TR\ Score = -8.132 + 1.226 * {Post - Tx}\ HCC\ EV\ Digital\ Score + 1.146 * \Delta\ HCC\ EV\ Digital\ Score$$

The boxplot (Fig. 6D) illustrated that the resulting HCC EV TR Scores were significantly higher in post-Tx viable HCC compared to post-Tx nonviable HCC (*P* < 0.0001). Overall, the HCC EV TR Score differentiated post-Tx viable HCC from nonviable HCC with an AUROC of 0.90 (95% CI = 0.82 – 0.99; sensitivity = 81.8%, specificity = 87.5%, accuracy = 83.7%; Fig. 6E) at the optimal cutoff (0.76) in the training set. The model, HCC EV TR Score, had an excellent calibration and discrimination capacity with c-statistic (0.88) to predict post-Tx viable HCC (Supplementary Fig. S6). Leave-one-out cross-validation of the training set confirmed the performance of the model (AUROC = 0.90, 95% CI = 0.82 – 0.99; sensitivity = 81.8%, specificity = 81.3%; Fig. 6F). To further assess model stability, we implemented tenfold cross-validation, which yielded an AUROC of 0.85 (Supplementary Fig. S7 A), and least absolute shrinkage and selection operator (LASSO) penalized regression, resulting in an AUROC of 0.83 (Supplementary Fig. S7B).

## Validation of HCC EV TR Scores for differentiating post-Tx viable and post-Tx nonviable HCC in the validation set

**Fig. 7:**
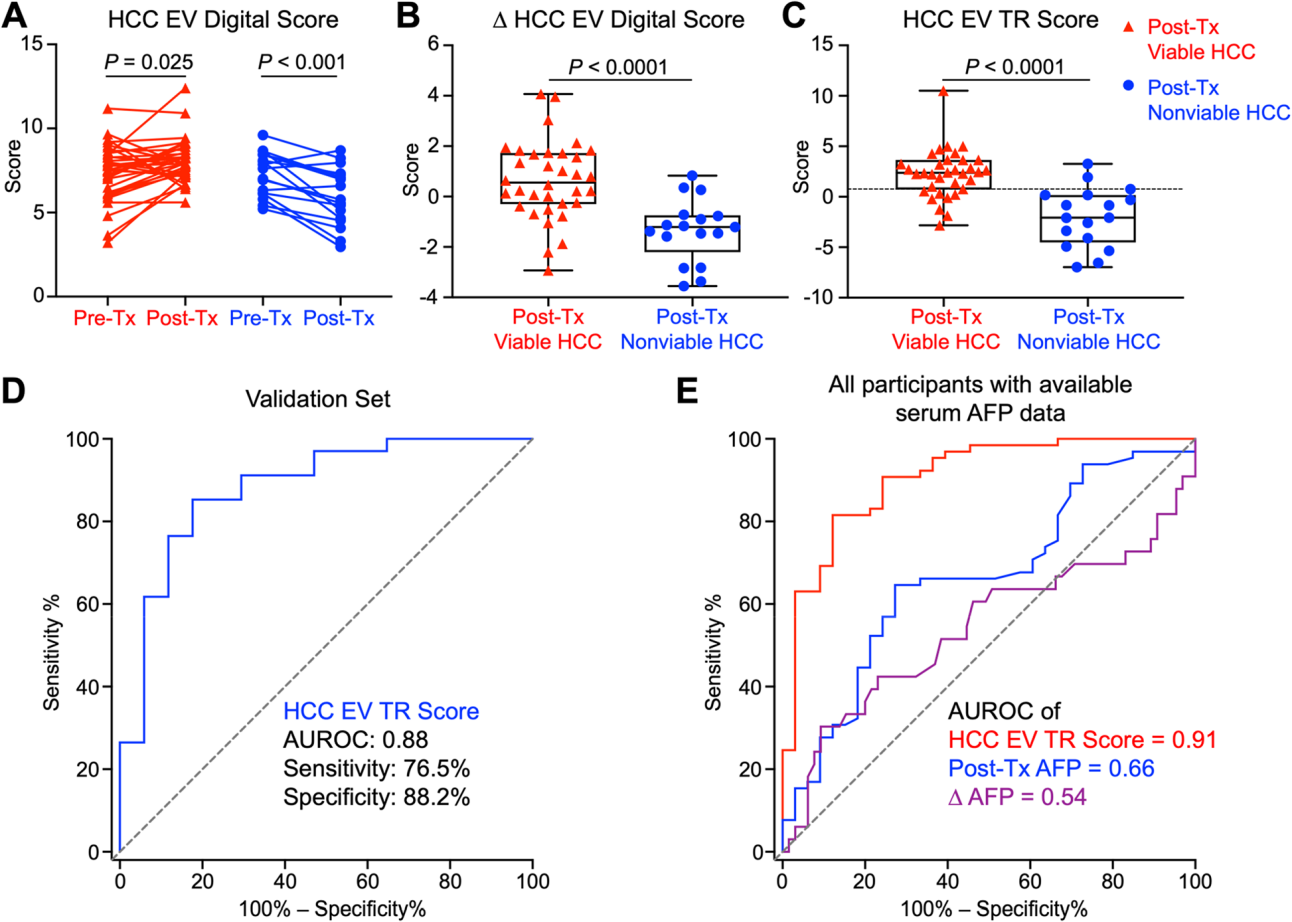
Validation of HCC EV TR Scores for differentiating post-Tx viable and post-Tx nonviable HCC in the validation set (*n* = 51). **A** Ladder plots summarizing the paired pre-Tx and post-Tx HCC EV Digital Scores for 34 post-Tx viable HCC patients and 17 post-Tx nonviable HCC patients. **B** Boxplots summarizing ∆ HCC EV Digital Scores for the 34 post-Tx viable HCC and 17 post-Tx nonviable HCC patients. **C** Boxplots showing the HCC EV TR Scores of the post-Tx viable and post-Tx nonviable HCC. The dashed line indicates the optimal cutoff of 0.76, calculated in the training set. **D** ROC curve of HCC EV TR Score for differentiating post-Tx viable HCC from post-Tx nonviable HCC in the validation set. **E** ROC curves of HCC EV TR Score, post-Tx serum AFP, and ∆ AFP in the combined training and validation set (98 patients with serum AFP data available). P = 0.0002 compared with post-Tx AFP and *P* < 0.0001 compared with ∆AFP using the paired DeLong’s test. AUROC, area under receiver operating characteristic curve; EV, extracellular vesicle; HCC, hepatocellular carcinoma; TR, treatment response; Tx, treatment

After obtaining the HCC EV TR Score formula from the training set, we further validated its performance in HCC TR assessment in a validation set with 51 patients including 34 post-Tx viable HCC and 17 post-Tx nonviable HCC cases. Figure 7A presents a ladder plot summarizing the paired pre-Tx and post-Tx HCC EV Digital Scores for the 51 HCC patients. As shown in Fig. 7B, post-Tx nonviable HCC showed significantly greater median decrease in ∆ HCC EV Digital Scores than post-Tx viable HCC. As shown in a boxplot (Fig. 7C), the post-Tx viable HCC exhibited significantly higher HCC EV TR Scores than post-Tx nonviable HCC (P < 0.001), resembling the patterns observed in the training set (Fig. 6D). In the validation set, HCC EV TR Scores exhibited high diagnostic performance to differentiate post-Tx viable versus nonviable HCC with an AUROC of 0.88 (95% CI: 0.78 – 0.98; Fig. 7D). At the cutoff value at 0.76 defined from the training set, HCC EV TR Score had great accuracy for detecting viable HCC with sensitivity of 76.5%, specificity of 88.2%, and accuracy of 80.4%. In the combined training and validation set comprising of 98 patients with serum AFP data available, the HCC EV TR Score outperformed serum AFP in distinguishing post-Tx viable from post-Tx nonviable HCC, with an AUROC of 0.91 (95% CI: 0.84 – 0.97; Fig. 7E). The performance of serum AFP in all populations (training set, validation set, all patients, and baseline AFP ≥ 10 ng/mL subpopulation), AUROC ranging from 0.51 to 0.69, was illustrated in Supplementary Fig. S8. Additionally, we evaluate the correlation between the six HCC EV specific genes and serum AFP, a well-known marker used for assessing tumor viability. The results showed that all six HCC EV-specific genes were not significantly correlated to serum AFP (Supplementary Fig. S9 A), underscoring the independent diagnostic value of these genes. The HCC EV TR Score was not correlated with serum AFP level, sex, liver disease etiology, or Tx options (Supplementary Fig. S9B-E), which suggested our results were not biased by these factors. Subgroup analyses were performed to evaluate the performance of the HCC EV TR Score across different treatment modalities and liver disease etiologies (Supplementary Fig. S10). The results demonstrated that the HCC EV TR Score remained robust in subgroups, with AUROCs ranging from 0.82 to 0.91, regardless of treatment approach or underlying liver disease etiology, reinforcing its broad clinical applicability.

## HCC EV TR Scores and clinical course of HCC patients

To illustrate the association between the calculated HCC EV TR Scores and the clinical course of each HCC patient, we created “integrated waterfall/swimmer plots” (Supplementary Fig. S11). These plots aim to showcase the remarkable diagnostic performance of the HCC EV TR Scores, alongside the LR-TR algorithm, in HCC patients treated with various therapeutic interventions. In the “integrated waterfall/swimmer plot” of the training set (Supplementary Fig. S11A), 81.8% of post-Tx viable HCC and 87.5% of post-Tx nonviable HCC corresponded to the clinical status determined by cross-sectional imaging data depicted in the swimmer plot. Similarly, 76.5% of post-Tx viable and 88.2% of post-Tx nonviable HCC patients showed concordance in the validation set (Supplementary Fig. S11B). Moreover, subgroup analysis also exhibited that the performance of HCC EV TR Score remained stable across different Tx options (Supplementary Table S3).

## Case study of post-Tx recurrence in six HCC patients with false negative cross-sectional imaging assessment and positive HCC EV TR Score readouts

**Fig. 8:**
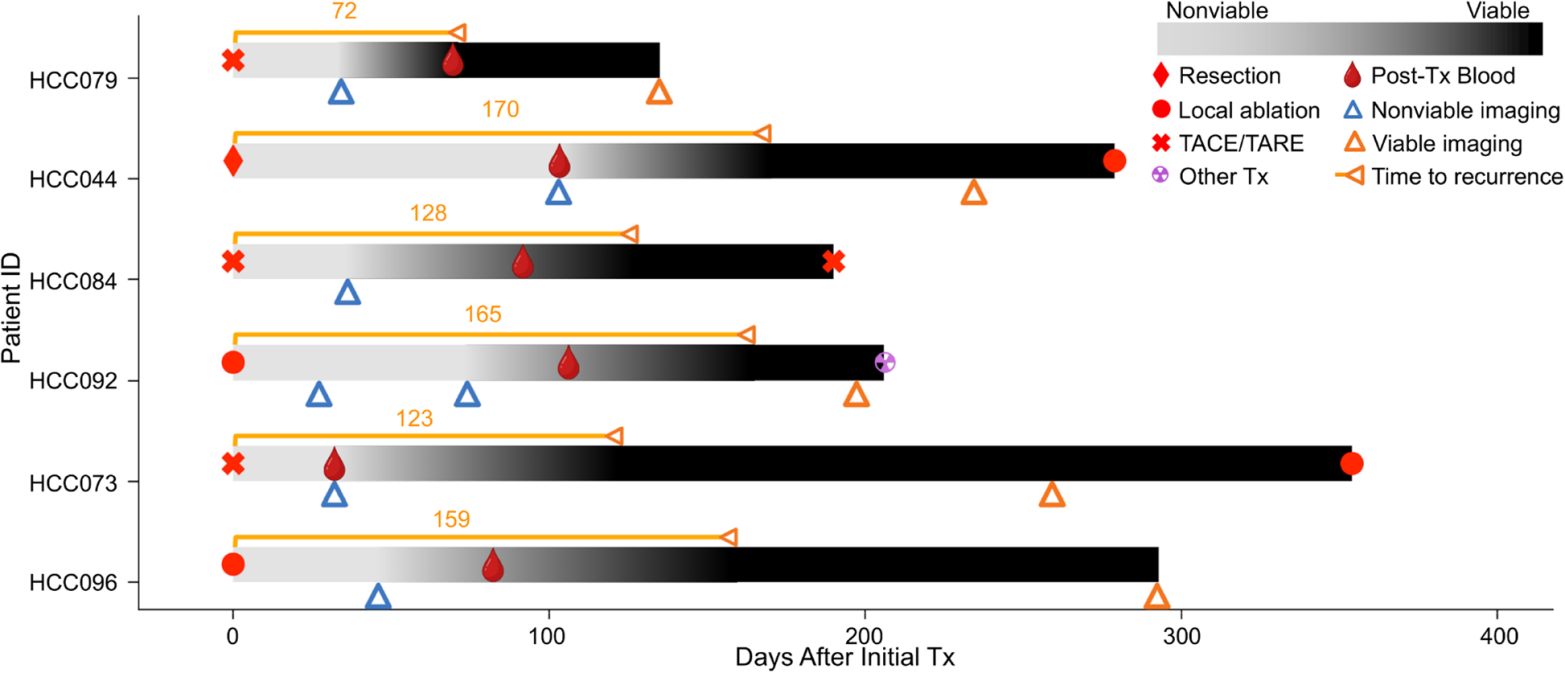
Case study of post-Tx recurrence in six HCC patients with false negative cross-sectional imaging assessment and positive HCC EV TR Score readouts. A swimmer plot illustrates the clinical histories of 6 HCC patients who exhibited discrepancies between their cross-sectional imaging assessment and HCC EV TR Score readouts. Each horizontal bar represents an individual patient's timeline from the initial Tx date (x-axis) to post-Tx recurrence based on cross-sectional imaging assessment. Blue triangles indicate the time points of initial cross-sectional imaging. Based on the LR-TR algorithm, all patients were classified as post-Tx nonviable HCC. Yet, their HCC EV TR Scores were above the optimal cutoff of 0.76, suggesting post-Tx viable status. Subsequent follow-up imaging confirmed the presence of viable lesions, consistent with the prediction by HCC EV TR Scores. TACE, transarterial chemoembolization; TARE, transarterial radioembolization; Tx, treatment

Among the 100 HCC patients enrolled in this study, 13 patients experienced early recurrence (defined as recurrence within 180 days post-Tx based on cross-sectional imaging assessment). Of these 13 patients, six exhibited discrepancies (Fig. 8) between their initial cross-sectional imaging (MRI) assessments and HCC EV TR Score readouts. They were initially classified as post-Tx nonviable HCC according to the LR-TR algorithm; however, their HCC EV TR Scores were above the optimal cutoff of 0.76, suggesting viable disease. Subsequent follow-up imaging confirmed the presence of viable lesions in all six cases with a median lead time of 63 days. These six clinical cases underscore the superior performance of HCC EV TR Scores, which could better predict the post-Tx recurrence in HCC patients and augment the conventional cross-sectional imaging to accurately distinguish post-Tx viable from post-Tx nonviable HCC (Detailed in Supplementary Note).

## Discussion

In this study, we developed and validated a two-step HCC EV Digital Scoring Assay for HCC TR assessment using 1.0-mL plasma samples from 100 early- or intermediate-stage HCC patients. The assay consists of EV Click Beads for HCC EV enrichment and RT-dPCR for absolute quantification of six HCC EV-specific genes. The superior specificity of HCC EV Digital Scoring Assay can be attributed to the two aspects. Firstly, the click chemistry-mediated HCC EV capture in the presence of HCC EV-associated antibody cocktail ensures the first layer of specificity of enriching HCC EVs [21]. Secondly, RT-dPCR were employed to detect HCC EV-specific genes (selected via the comprehensive data analysis pipeline) in the enriched HCC EVs, conferring the second layer of specificity to the assay. From the resulting 6-gene readouts, we obtained pre-Tx, post-Tx, and ∆ HCC EV Digital Scores for each patient. By synergistically combining the post-Tx and ∆ HCC EV Digital Scores, we developed the model for HCC TR assessment, i.e., HCC EV TR Score, in the training set (AUROC of 0.90). The performance of HCC EV TR Score to distinguish post-Tx viable from post-Tx nonviable HCC was reproduced in the validation set, with an AUROC of 0.88. We created “integrated waterfall/swimmer plots” to summarize the correspondence between the resultant HCC EV TR Scores of the 100 enrolled HCC patients and their respective longitudinal clinical histories, revealing the performance of HCC EV TR Scores to distinguish post-Tx viable from post-Tx nonviable HCC. HCC EV TR Score also identified residual disease not initially observed on MRI in six patients, with a median lead time of 63 days. Collectively, this EV-based digital scoring approach [21, 36] shows a great potential in augmenting current cross-sectional imaging techniques for clinical decision making.

The comprehensive data analysis pipeline developed in this study is an innovative approach that combines multiple steps to identify and validate HCC EV-specific gene candidates and assess TR in HCC patients. The pipeline leverages a comprehensive database (LiTA) created by aggregating publicly available liver transcriptome data and incorporates diverse datasets from different sources. To our knowledge, LiTA is the most comprehensive transcriptome database for HCC and liver disease, which covers all common etiologies of chronic liver diseases, including HBV, HCV, ALD, and MASLD. It employs a multi-step selection process to ensure the specificity of the identified genes to HCC EVs. Of note, we applied the exoRBase 2.0 [31], an EV-based dataset, as the filter to select the candidate markers. This approach helps translate the tissue-based transcriptome into an EV-based setting, reducing the discrepancy of the expression levels between the different sources and paving the way for our subsequent validation. The pipeline also integrated validation using RNAscope, ensuring that these selected HCC EV-specific gene candidates are differentially expressed in HCC over the matched negative surgical margins.

The selected six HCC EV-specific genes, i.e., *ALB*, *APOH*, *FGB*, *FGG*, *H2AX*, and *TF*, were identified based on their well-established roles in HCC biology and their potential for noninvasive detection of HCC. *ALB* and *APOH*, frequently altered in HCC, contribute to metabolic reprogramming, a hallmark of tumor progression [37]. The fibrinogen subunits *FGB* and *FGG* play essential roles in the tumor microenvironment by promoting thrombosis, which has been linked to HCC progression [38]. *H2AX*, a critical component of the DNA damage response pathway, is associated with HCC radioresistance [39], while *TF*, a key regulator of iron metabolism, influences tumor growth and oxidative stress in HCC [40]. Beyond their biological relevance, five of these genes (*ALB*, *APOH*, *FGB*, *FGG*, and *TF*) have been previously validated in HCC EVs for early detection [21] and have also been detected in circulating tumor cells (CTCs), underscoring their relevance as biomarkers for noninvasive HCC diagnostics and treatment monitoring [32]. The integration of these HCC EV-specific genes into our digital scoring approach further strengthens its potential for improving TR assessment and enhancing liquid biopsy applications in HCC management.

Cross-sectional imaging, such as CT and MRI, is the cornerstone of HCC TR assessment, offering detailed visualization of tumor size, location, and vascular involvement [41]. Despite its advantages, cross-sectional imaging has limitations, including potential inaccuracies in distinguishing viable from nonviable tumor tissue in the setting of short-term post-Tx changes, radiation exposure, psychological stress, variability in interpretation and imaging protocols, and high costs [42], especially in the patients with equivocal results who require repeated tests. These challenges highlight the need for complementary noninvasive tools such as liquid biopsy to enhance accuracy and early detection of residual disease. Liquid biopsy approaches utilizing total EVs, circulating tumor DNA and circulating tumor cells have been explored for assessing HCC TR (Table S4) [43–46]. However, these methods lack validation phase. Therefore, a well-developed and clinically validated approach remains necessary to ensure reliability and clinical applicability. As one of the emerging liquid biopsy tools, tumor-derived EVs are detectable in circulation in early stage of disease [47], and their mRNA cargoes has been shown to be highly stable in plasma over extended periods when stored at − 80 °C [48, 49]. These advantages underscore their appeal for cancer biomarker development, especially in multicenter clinical trials where plasma samples need to be stored and shipped across different institutions [50]. On the basis of our developed HCC EV assays [21, 22], we selected the gene panel and expanded the utility of HCC EV Digital Scoring Assay to HCC TR assessment. The resulting HCC EV TR Score demonstrated enhanced sensitivity and specificity by synergistically combining post-Tx HCC EV Digital Score and ∆ HCC EV Digital Score. Higher post-Tx HCC EV Digital Scores reflect the presence of residual tumor and HCC EVs, enhancing sensitivity for detecting viable disease. Conversely, negative Δ HCC EV Digital Scores indicate a decrease from pre-Tx to post-Tx Scores, reflecting the decrease of residual tumor and HCC EVs, thus improving specificity for detecting nonviable disease. The HCC EV TR Score leverages the strengths of both components, providing a robust tool for accurately assessing TR in HCC. More importantly, the performance of HCC EV TR Score remained stable in the validation set and across different Tx options, showing the credibility of the assay and paving the way for further validation. The effectiveness of the HCC EV TR Score in the early detection of residual disease in HCC patients is pivotal for refining Tx strategies. This capability may facilitate the initiation of earlier interventions, including the administration of adjuvant therapies and potential addition of systemic therapies, thereby enhancing patient management and outcomes.

Despite the promising findings, this study has several limitations. First, as a retrospective phase-2 biomarker study, it inherently carries biases related to patient selection and data collection. Second, the limited sample size constrained our ability to conduct more granular subgroup analyses, which could have provided deeper insights into specific patient subsets. Future research will involve larger-scale, prospective studies with extensive external validation and a sufficient number of explant pathological specimens to definitively confirm post-Tx viable or nonviable tumor status in Tx-specific subgroups. These efforts will be crucial in solidifying the clinical utility of the HCC EV TR Score for TR assessment in HCC.

In conclusion, we developed and validated the HCC EV TR Score based on HCC EV Digital Scoring Assay for accurate differentiation of post-Tx viable from nonviable HCC cases. The HCC EV TR Score demonstrated outstanding sensitivity and specificity with consistently excellent performance in both the training and validation sets. This liquid biopsy approach holds great promise for earlier detection of recurrence in HCC, augmentation of current cross-sectional imaging assessments, and assistance in clinical decision-making for HCC patients.

## Supplementary Information

Supplementary Material 1.

## Data Availability

The data utilized in this study were sourced from the GEO and are publicly accessible in the National Center for Biotechnology Information's GEO database (https://www.ncbi.nlm.nih.gov/geo/), as well as the ArrayExpress database hosted by the European Bioinformatics Institute (EBI) (https://www.ebi.ac.uk/biostudies/arrayexpress). Reagents employed in this investigation are comprehensively presented in the Supplementary materials accompanying this manuscript. Further elucidation on the protocols and statistical code employed in this study can be obtained from the corresponding authors upon request.

## Abbreviations

AFP: Alpha-fetoprotein
ASGPR1: Asialoglycoprotein receptor 1
AUROC: Area under the receiver operating characteristic curve
EpCAM: Epithelial cellular adhesion molecule
EV: Extracellular vesicles
HCC: Hepatocellular carcinoma
LiTA: Liver Transcriptome Atlas
LT: Liver transplantation
mTz: Methyltetrazine
ROC: Receiver operating characteristic
RT-dPCR: Reverse-transcription digital polymerase chain reaction
TACE: Transarterial chemoembolization
TARE: Transarterial radioembolization
TCO: Trans-cyclooctene
TR: Treatment response
Tx: Treatment
