# Supplementary material for: Extracellular vesicle digital scoring assay for assessment of treatment responses in hepatocellular carcinoma patients
Source: J Exp Clin Cancer Res. 2025 May 1;44:136. doi: 10.1186/s13046-025-03379-7 (PMC12044846; doi:10.1186/s13046-025-03379-7)
Supplement: Supplementary file 1 — Supplementary Material 1. [file 13046_2025_3379_MOESM1_ESM.pdf]

# Supplementary Information

# Extracellular vesicle digital scoring assay for assessment of treatment responses in hepatocellular carcinoma patients

Chen Zhao[1, 2, 3], Yi-Te Lee[4], Andrew Melehy[5], Minhyung Kim[6], Jacqueline Ziqian Yang[1,2], Ceng Zhang[2], Jina Kim[6], Ryan Y. Zhang[2], Junseok Lee[2], Hyoyong Kim[2], Yong Ju[2],Yuan-Jen Tsai[2, 7], Xianghong Jasmine Zhou[1], Steven-Huy B. Han[8], Saeed Sadeghi[8], Richard S. Finn[8], Sammy Saab[8], David S. Lu[9], Jason Chiang[9], Jae-Ho Park[4], Todd V. Brennan[10, 11], Steven A. Wisel[10, 11], Manaf Alsudaney[11], Alexander Kuo[4,11], Walid S. Ayoub[4, 11], Hyunseok Kim[4, 11], Hirsh D. Trivedi[4, 11], Yun Wang[4, 11], Aarshi Vipani[4, 11], Irene K. Kim[11], Tsuyoshi Todo[11], Justin A. Steggerda[11], Georgios Voidonikolas[11], Kambiz Kosari[11], Nicholas N. Nissen[11], Rola Saouaf[12], Amit G. Singal[13], Myung Shin Sim[14], David A. Elashoff[8], Sungyong You[6,15]*, Vatche G. Agopian[5,16]*, Ju Dong Yang[4,11,15]*, Hsian-Rong Tseng[2,16]*, and Yazhen Zhu[1,2,16]*

1 Department of Pathology and Laboratory Medicine, David Geffen School of Medicine, University of California, Los Angeles (UCLA), Los Angeles, CA 90095, USA
2 California NanoSystems Institute, Crump Institute for Molecular Imaging, Department of Molecular and Medical Pharmacology, University of California, Los Angeles (UCLA), Los Angeles, CA 90095, USA
3 Cancer Center, Renmin Hospital of Wuhan University,430060 Wuhan, P.R. China
4 Karsh Division of Gastroenterology and Hepatology, Cedars-Sinai Medical Center, Los Angeles, CA 90048, USA
5 Department of Surgery, David Geffen School of Medicine, University of California, Los Angeles (UCLA), Los Angeles, CA 90095, USA
6 Department of Urology and Computational Biomedicine, Cedars-Sinai Medical Center, Los Angeles, CA 90048, USA
7 Department of Family Medicine, Taipei Medical University Hospital, Taipei 110301, Taiwan
8 Department of Medicine, David Geffen School of Medicine, University of California, Los Angeles (UCLA), Los Angeles, CA 90095, USA
9 Department of Interventional Radiology, David Geffen School of Medicine, University of California, Los Angeles (UCLA), Los Angeles, CA 90095, USA
10 Department of Surgery, Cedars-Sinai Medical Center, Los Angeles, CA 90048, USA
11 Comprehensive Transplant Center, Cedars-Sinai Medical Center, Los Angeles, CA 90048, USA
12 Department of Radiology, Cedars-Sinai Medical Center, Los Angeles, CA 90048, USA
13 Division of Digestive and Liver Diseases, Department of Internal Medicine, University of Texas Southwestern Medical Center, Dallas, TX 75390, USA.
14 Department of Medicine Statistics Core, David Geffen School of Medicine, University of California, Los Angeles (UCLA), Los Angeles, CA 90095, USA
15 Samuel Oschin Comprehensive Cancer Institute, Cedars-Sinai Medical Center, Los Angeles, CA 90048, USA
16 Jonsson Comprehensive Cancer Center, University of California, Los Angeles (UCLA), Los Angeles, CA 90095, USA

*Corresponding author. Email: sungyong.you@cshs.org (S.Y.); vagopian@mednet.ucla.edu (V.G.A.); judong.yang@cshs.org (J.Y.); hrtseng@mednet.ucla.edu (H.R.T.); yazhenzhu@mednet.ucla.edu (Y.Z.).

## Supplementary Methods

### Patient enrollment

Liver cirrhosis was identified based on one or more of the following criteria: histological features consistent with cirrhosis, imaging characteristics indicative of cirrhosis (such as a nodular liver surface) or portal hypertension (evidenced by splenomegaly or portosystemic collaterals), MR elastography, or FibroScan® assessment. Eligible cirrhosis patients required either a minimum of 12 months follow-up post-enrollment or a recent CT or MRI scan (within two weeks of blood collection) confirming the absence of hepatocellular carcinoma (HCC). HCC was diagnosed according to the American Association for the Study of Liver Diseases (AASLD) clinical practice guideline [1, 2]: (i) histology or (ii) CT or MRI imaging categorized as Liver Imaging Reporting and Data System (LI-RADS) 5.

### Definition of underlying liver disease

Etiologies of underlying liver disease were defined as previous studies [3]. In brief, chronic hepatitis B virus (HBV) infection was diagnosed by positive HBsAg. Chronic hepatitis C virus (HCV) infection was diagnosed by positive HCV RNA results or chronic liver disease with positive anti-HCV. Alcoholic liver disease (ALD) was diagnosed by a documented history of ALD or significant alcohol abuse or addiction. Metabolic dysfunction-associated steatotic liver disease (MASLD) was diagnosed following the AASLD MASLD diagnostic criteria [4].

### Comprehensive data analysis pipeline for identifying the 12 HCC EV-specific gene candidates

To identify the HCC extracellular vesicle (EV)-specific gene candidates, we implemented a data analysis pipeline utilizing the Liver Transcriptome Atlas (LiTA). The LiTA comprises a comprehensive set of transcriptome data from liver tissues affected by various etiologies, including viral infections and metabolic disorders, covering healthy tissues, benign liver diseases, cirrhosis, and HCC. Using the LiTA, we selected HCC EV-specific genes (**Figure 2**, right). Initially, we identified genes that were highly expressed (top 5% of fold change) and exhibited a false discovery rate (FDR) of less than 0.05. These genes underwent four comparative analyses based on etiology: (i) HCC vs cirrhosis, (ii) HCV-related HCC vs HCV-related cirrhosis, (iii) HBV or hepatitis D virus (HDV)-related HCC vs HBV or HDV-related non-HCC, and (iv) ALD, MASLD, or other disorder-related HCC vs corresponding cirrhosis. From these upregulated genes, we selected those

with a median expression value greater than five in HCC cell lines, which is the cutoff value of absent/present calling, using the Cancer Cell Line Encyclopedia (CCLE) dataset [5]. To minimize background signals from blood-origin EVs, we excluded genes with a maximum expression value exceeding 10.04 (top 40%) in immune cells, using the Differentiation MAP dataset (DMAP) [6]. To further refine the HCC EV marker gene set, we integrated RNA sequencing data from the exoRBase 2.0 database [7] to pinpoint genes highly expressed in EVs derived from HCC patients. The overlap between the LiTA results and the exoRBAse 2.0 data yielded 60, 12, 45, and 102 potential HCC EV marker gene candidates from the four comparative analyses, respectively. From these, we selected the top 16 HCC EV-specific gene candidates that could differentiate HCC and cirrhosis (*VPS72*, *TMEM106C*, *PRIM1*, *SPDL1*, *FLAD1*, *SMYD3*, *SORT1*, *ATAD2*, *SETDB1*, *H2AX*, *PUF60*, *TUBG1*, *STT3A*, *UBL4A*, *C8orf33*, and *KLHL12*).

**RNAScope image acquisition and analysis**

RNAScope images were captured using the Aperio VERSA Brightfield, Fluorescence & FISH Digital Pathology Scanner at the Translational Pathology Core Laboratory (TPCL) of University of California, Los Angeles (UCLA). Initial acquisition settings were standardized and maintained consistently. Color deconvolution was applied, and excitation channels were designated for detecting nuclear counterstains and mRNA probes (DAPI, 405 nm; Cy5 for CD147, 690 nm; mRNA probe, 570 nm). Images were pre-processed using Image J and analyzed on QuPath [8] following the QuPath Analysis Guidelines. Cell segmentation was performed using the Analyze-Cell detection module, and subcellular detection module was employed for probe detection. The count of single spots was tallied for the quantification of test probes [9]. Single spots were counted for probe quantification, with the threshold set at the mean number of single spots in the negative control probe (mean = 1). Cells were classified based on probe signal intensity: negative (≤1 spot), 1+ (2-4 spots), 2+ (5-9 spots), and 3+ (≥10 spots).

**Cell culture**

HCC cell line HepG2 (ATCC HB-8065) was cultured in Eagle's Minimum Essential Medium (EMEM, Thermo Fisher Scientific, USA) supplemented with 10% fetal bovine serum and 100 U/mL penicillin-streptomycin. Cultures were maintained in a humidified incubator at 37°C with 5% $CO_2$. To harvest HCC cell line-derived EVs, HepG2 cells were grown to 70% confluence in 18 Nunc™ EasYDish™ Dishes (145 $cm^2$, Thermo Fisher Scientific, USA) and subsequently

incubated in serum-free media for 24-48 hours. The conditioned serum-free media was then collected for EV isolation.

**Harvest of HepG2 EVs by ultracentrifugation**

The conditioned serum-free media was first centrifuged at 300g (4°C) for 5 minutes to pellet cells, repeated three times. The supernatant was centrifuged at 2,800g (4°C) for 10 minutes to remove residual cell debris. The resulting supernatant was transferred to Open-Top Thinwall Ultra-Clear Tubes (Beckman Coulter, USA) and ultracentrifuged at 100,000g (4°C) for 70 minutes using an SW 32 Ti rotor in an Optima L-100 XP Ultracentrifuge (Beckman Coulter, USA). Finally, the HCC EV pellet was resuspended in 200 µL ice-cold phosphate-buffered saline (PBS) and aliquoted for downstream applications.

**Nanoparticle tracking analysis (NTA)**

EV size distributions and particle concentrations were characterized utilizing NTA on the Zetasizer Nano ZS instrument (Malvern Instruments Ltd., UK). Prior to NTA measurements, EV samples were diluted to either a 1:10 or 1:20 ratio in PBS to achieve optimal vesicle concentration and minimize multiple scattering effects. The diluted EV samples were loaded into low-volume disposable cuvettes and equilibrated to 25°C inside the Zetasizer instrument. NTA data was acquired and analyzed using Zetasizer software to generate size distribution profiles.

**Transmission electron microscopy (TEM)**

10-µL HepG2 EVs were treated with 4% paraformaldehyde (PFA) for 30 minutes and deposited onto 200-mesh formvar-carbon coated EM grids for 20 minutes. The grids were washed with water drops for 5 times and then exposed to 1% uranyl acetate for 1 minute. Excessive fluid was removed by blotting with filter paper, and the grids were left to air dry. Finally, imaging of these grids was performed using a Tecnai 12 Quick Cryo-Electron Microscope (FEI, USA).

For immunogold staining, HepG2 EV samples were initially enriched using EV Click Beads that had been grafted with monoclonal mouse IgG human CD63 antibody (diluted at 1:100, R&D systems) for 30 minutes. Subsequently, the samples were incubated with anti-mouse nanogold (12 nm, diluted at 1:50) for 1 hour. The gold-labeled EV samples were then applied onto carbon-coated copper grids and allowed to incubate for 10 minutes before being wiped off the grids. Following this step, the grids were rinsed five times with water and then dried before TEM imaging.

**Linearity assessment of HCC EV Digital Scoring Assay**

To evaluate the linearity and dynamic range of HCC EV Digital Scoring Assay with 12 HCC EV-specific gene candidates, synthetic plasma samples were prepared by serially spiking HepG2 EVs (10 µL) into EV-depleted plasma from healthy donor (90 µL). 0.1-mL synthetic plasma were incubated and labeled with a 14-µL antibody cocktail containing TCO-anti-EpCAM (200 ng), TCO-anti-CD147 (250 ng), and TCO-anti-ASGPR1 (500 ng), followed by EV enrichment using EV Click Beads. As negative controls, identical experiments were performed with spiking fetal bovine serum (FBS). After EV enrichment using EV Click Beads, the EV cargo RNA was extracted and absolute quantification of 12 HCC EV-specific gene candidates was performed by reverse transcription-digital PCR (RT-dPCR). All samples were prepared in duplicate.

**Statistics**

The Mann-Whitney U test followed by the Benjamini-Hochberg false discovery rate (FDR) correction were performed to identify highly expressed genes in HCC compared to cirrhosis using the LiTA. Linear regression analysis was conducted to assess the linearity correlations between the concentration of spiked HepG2 EVs and measured mRNA copy numbers in synthetic plasma samples by calculating the slope and coefficient of determination ($R^2$).

For model validation, we employed a comprehensive approach using 10-fold cross-validation and LASSO penalized regression. A calibration plot of HCC EV TR Score was generated to investigate the agreement between the predicted probabilities of post-Tx viable HCC and the observed outcomes. The C-statistic was used to measure the discrimination between predicted probabilities and the observed outcomes. Correlation between HCC EV TR Score and serum AFP level was determined by Spearman's correlation. The Mann-Whitney U test and The Kruskal-Wallis test were employed to compare differences of HCC EV TR Score in categorical variables.

## Supplementary Note

### Case Study: Post-Tx Recurrence in HCC Patients with False Negative Imaging and Positive HCC EV TR Score Readouts

In the six cases of HCC, discrepancies were observed between initial cross-sectional imaging assessments and HCC EV TR Score results, as illustrated in **Figure 7**. These cases highlight the potential of the HCC EV TR Score to detect residual disease earlier than conventional imaging.

**Case HCC079**: The patient is a 58-year-old male with BCLC stage B HCC who underwent transcatheter arterial chemoembolization (TACE). The post-Tx MRI conducted at 34 days after TACE suggested a nonviable tumor. However, the HCC EV TR Score derived from the post-Tx blood draw at 69 days after TACE indicated a viable disease and a treated residual viable lesion was later confirmed by MRI at 72 days post-Tx (The short-term follow-up imaging was performed due to a motion artifact noted in the previous MRI). In this case, the HCC EV TR Score had a leading time of 3 days compared to subsequent follow-up imaging.

**Case HCC044**: The patient is a 58-year-old male with BCLC stage B HCC who underwent an extended left hepatectomy. Initial post-Tx MRI at 103 days post-surgery indicated no viable tumor, yet the HCC EV TR Score derived from the same day post-Tx blood draw revealed the presence of viable disease. Follow-up imaging at 170 and 234 days post-surgery identified a new 1.0 cm lesion in segment 7, leading to subsequent ablation therapy. In this case, the HCC EV TR Score had a lead time of 67 days compared to subsequent follow-up imaging.

**Case HCC084**: The patient is a 68-year-old male with BCLC stage A HCC and treated with TACE. MRI at 36 days post-Tx classified the tumor as nonviable, but the HCC EV TR Score from the blood draw at 92 days post-Tx suggested a viable tumor. Subsequent imaging at 128 days post-Tx confirmed the presence of two viable lesions (a treated residual lesion and a new lesion), which makes the lead time of HCC EV TR Score 36 days earlier than subsequent follow-up imaging.

**Case HCC092**: The patient is a 72-year-old female with BCLC stage A HCC who underwent microwave ablation (MWA) of a 1cm HCC tumor. MRI assessment at 27 and 74 days post-Tx classified the tumor as nonviable. However, the HCC EV TR Score derived from the blood draw 106 days post-Tx indicated viable disease, which was later confirmed by imaging at 165 and

197 days post-Tx. Immunotherapy was then administered to treat the new lesion. In this case, the HCC EV TR Score had a lead time of 59 days compared to subsequent follow-up imaging.

**Case HCC073**: The patient is a 68-year-old male with BCLC stage A HCC and was treated with transarterial radioembolization (TARE) using Y90. MRI at 32 days post-Tx indicated a nonviable tumor, but the HCC EV TR Score derived from the same day post-Tx blood draw suggested viable tumor. Subsequent imaging at 123 and 259 days post-Tx confirmed a new lesion, which led to the addition of MWA therapy. In this case, the HCC EV TR Score had a lead time of 91 days compared to subsequent follow-up imaging.

**Case HCC096**: The patient is a 60-year-old female with BCLC stage A HCC who underwent MWA therapy for the 2.3 cm tumor. MRI at 46 days post-Tx classified the tumor as nonviable. However, the HCC EV TR Score derived from a blood draw 82 days post-Tx indicated viable disease and was then confirmed to be a recurrent lesion by imaging at 159 and 293 days post-Tx. In this case, the HCC EV TR Score had a lead time of 77 days compared to subsequent follow-up imaging.

These six cases illustrate the superior performance of the HCC EV TR Score in predicting post-Tx recurrence in HCC patients and augmenting conventional cross-sectional imaging to more accurately detect post-Tx viable and nonviable HCC.

# Supplementary Figures

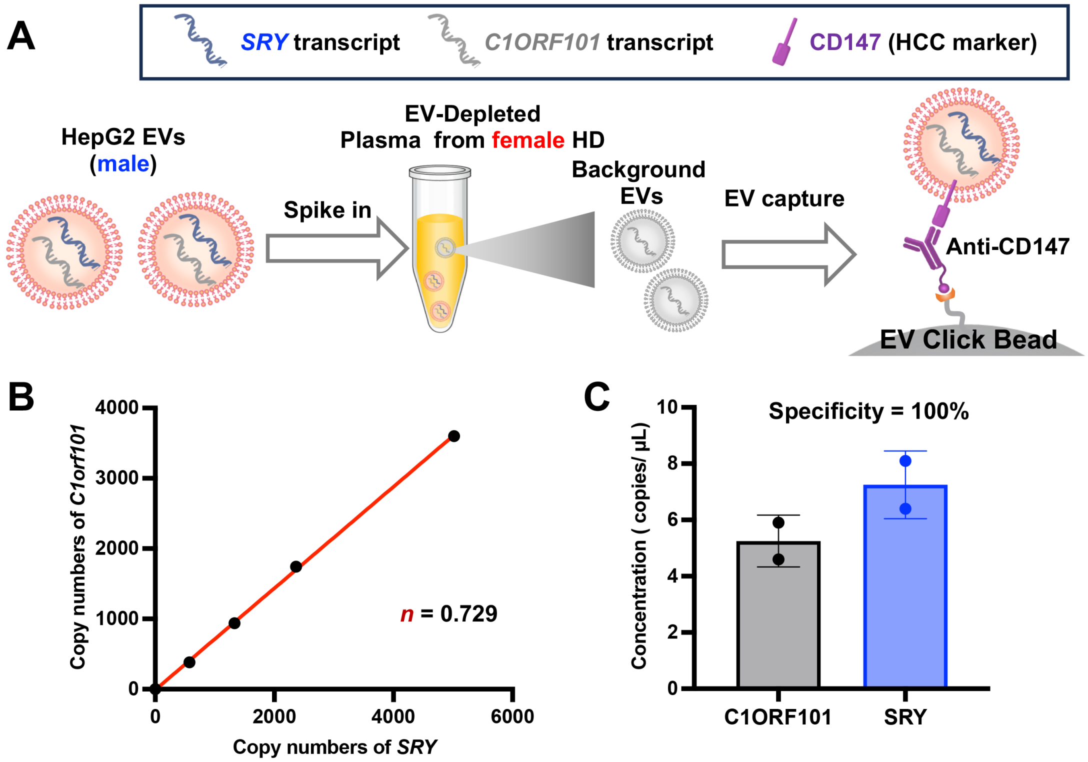


$$n = \frac{\textit{C1ORF101}\text{ transcripts}_{\text{input EV}}}{\textit{SRY}\text{ transcripts}_{\text{input EV}}} \qquad \text{Specificity} = \frac{n \times \textit{SRY}\text{ transcripts}_{\text{enriched EV}}}{\textit{C1ORF101}\text{ transcripts}_{\text{enriched EV}}}$$

**Fig. S1. HCC EV capture specificity was calculated by quantifying *SRY* gene in HepG2 EV spiked into female HD samples.**
**(A**) Synthetic plasma samples were prepared by spiking 10-µL aliquoted HepG2 cell-derived EVs into 90-µL EV-depleted plasma from a female healthy donor (HD). The plasma samples were then incubated with the EV Click Beads with the presence of TCO-anti-CD147 for HCC EVs enrichment. RT-dPCR was employed to quantify the copy numbers of the *SRY* transcripts and *C1ORF101* transcripts in the enriched EV samples to calculate the specificity. *SRY* transcripts (which stand for sex-determining region Y gene) were contributed by HepG2 EVs while *C1ORF101* transcripts were contributed by both enriched HepG2 EVs and the non-specifically captured background female plasma-derived EVs. **(B)** Verification of the linear correlation between copy numbers of *C1orf101* and *SRY* transcripts in pure HepG2 EVs. The ratios between *C1orf101* and *SRY* transcripts in HepG2 EVs exhibited a consistent linear correlation (y = 0.729 x, $R^2$ = 0.999) with the *C1orf101*-to-*SRY* ratio determined as 0.729. **(C)** The specificity of HepG2 EV enrichment from EV Click Beads were calculated as the ratio of enriched *SRY* to *C1orf101* transcripts. $n$ = 0.729 is specific to HepG2 EVs in this study. Data are presented as means ± standard deviation.

EV, extracellular vesicle; HCC, hepatocellular carcinoma; HD, healthy donor; RT-dPCR, reverse-transcription digital PCR.

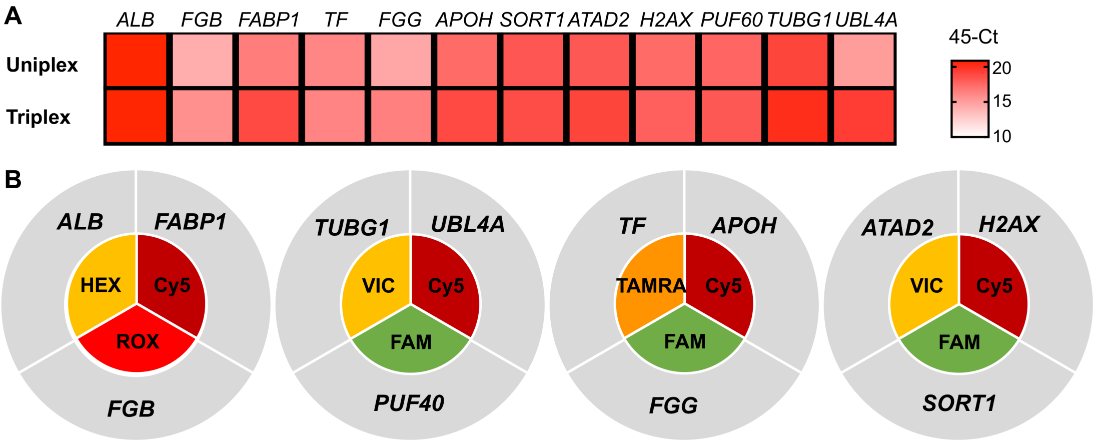


**Fig. S2. Validation of primers and probes for the 12 HCC EV-specific gene candidates.**
**(A)** Signals for the 12 HCC EV-specific gene candidates were compared in uniplex and triplex quantitative PCR (qPCR) using 2.0 ng of cDNA from HepG2 cells. **(B)** Schematic representation of the triplex qPCR design, with three genes assigned to each PCR tube for the 12 HCC EV-specific gene candidates.

EV, extracellular vesicle; HCC, hepatocellular carcinoma.

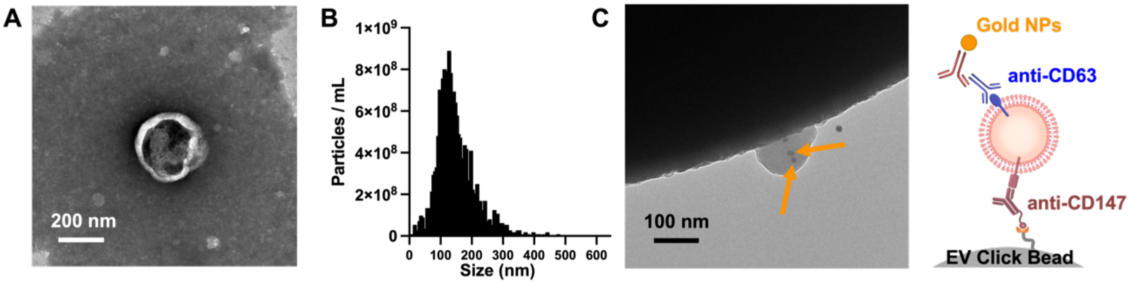


**Fig. S3. Characterization of HepG2 extracellular vesicles (EVs).**

**(A)** A representative transmission electron microscope (TEM) image of HepG2 EVs. **(B)** Size distribution of HepG2 EVs measured by and nanoparticle tracking analysis (NTA), suggesting that the sizes of these HepG2 EVs were 133.2 ± 60.9 nm. **(C)** A representative high-resolution TEM image of HepG2 EVs immobilized on an EV Click Bead followed by immunogold labelling of CD63, a representative EV surface marker. After capturing the HepG2 EVs, the presence of multiple gold nanoparticles was observed. Scale bar, 100 nm. Yellow arrows indicate gold nanoparticles.

EV, extracellular vesicle; NP, nanoparticle

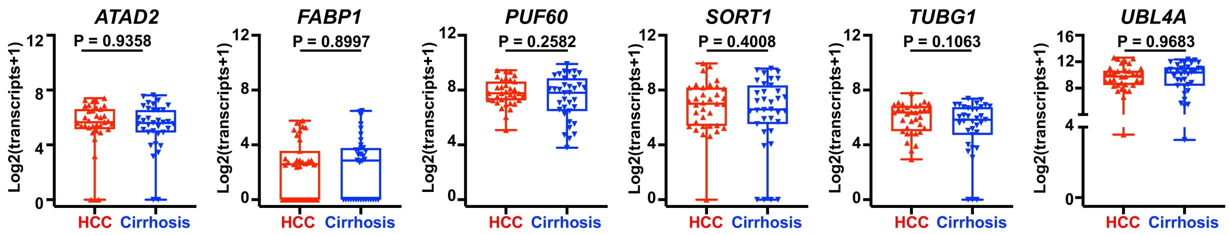


**Fig. S4. Expression levels of the six non-differential HCC EV-specific genes.**
The expression levels of six HCC EV-specific genes (*ATAD2*, *FABP1*, *PUF60*, *SORT1*, *TUBG1*, and *UBL4A*), which showed no significant differences between 35 early-stage HCC patients and 35 cirrhosis patients.

EV, extracellular vesicle; HCC, hepatocellular carcinoma.

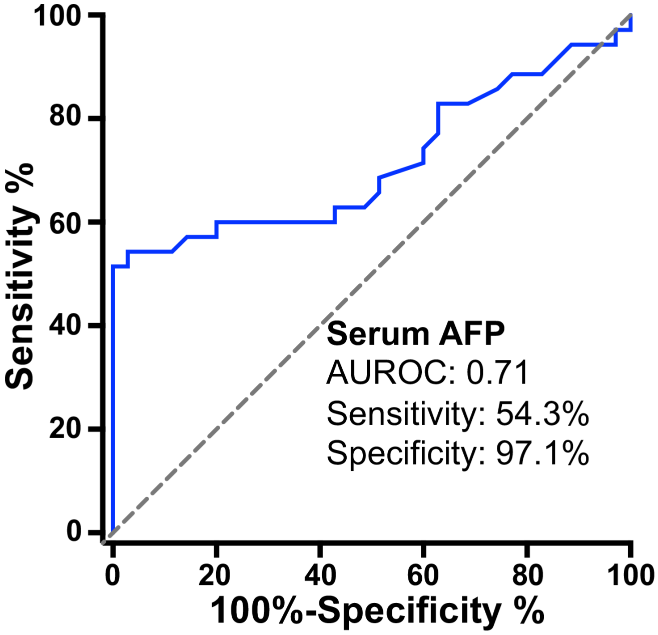


**Fig. S5. Receiver operating characteristic (ROC) curve of serum alpha-fetoprotein (AFP) for distinguishing early- or intermediate-stage HCC (*n* = 35) from liver cirrhosis (*n* = 35).**

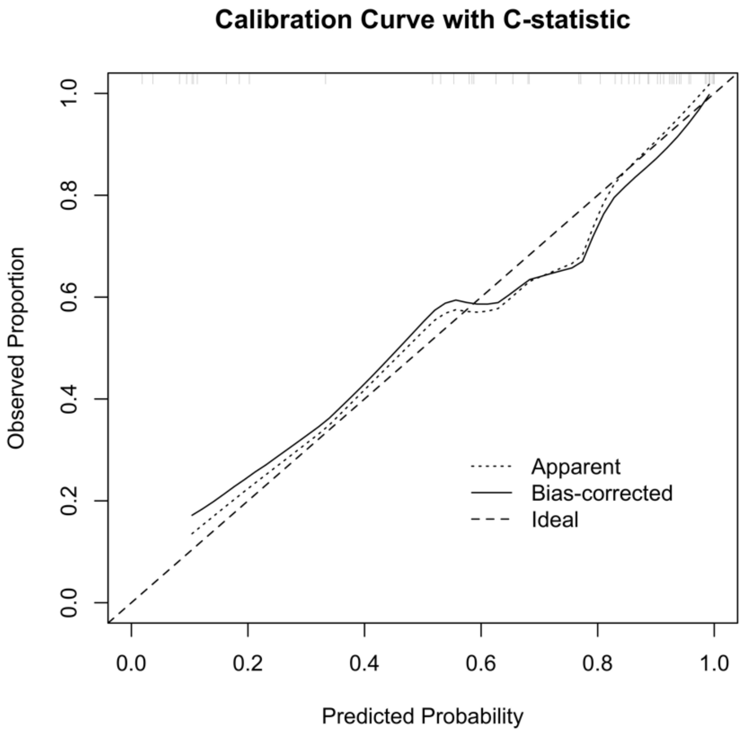


**Fig. S6. Calibration plot of HCC EV TR Score.**
A calibration curve of HCC EV TR Score shows the predicted probability of post-Tx viable HCC in the training set of 49 HCC patients, demonstrating good conformity to the actual probability with a high c-statistic (0.88).

EV, extracellular vesicle; HCC, hepatocellular carcinoma; TR, treatment response; Tx, treatment.

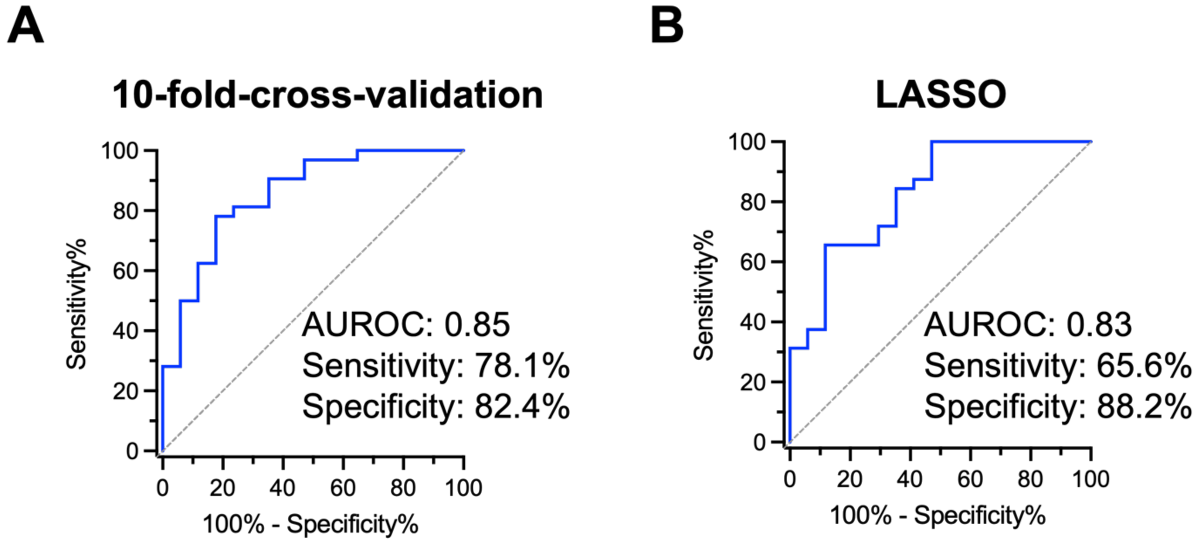


**Fig. S7. Model validation using 10-fold cross-validation and least absolute shrinkage and selection operator (LASSO) penalized regression. (A)** A 10-fold cross-validation resulted in an AUROC of 0.85, with sensitivity of 78.1% and specificity of 82.4% at optimal cutoff. **(B)** LASSO penalized regression yielded an AUROC of 0.83 with sensitivity of 65.6% and specificity of 88.2% at optimal cutoff. The slight variation in AUROC (ranging from 0.83 to 0.90) across these validation methods indicates that our model maintains strong predictive performance without significant overfitting. The trade-offs between sensitivity and specificity observed in the LASSO model (higher specificity at the cost of lower sensitivity) are expected due to the regularization applied in feature selection.

AUROC, area under the receiver operating characteristic curve

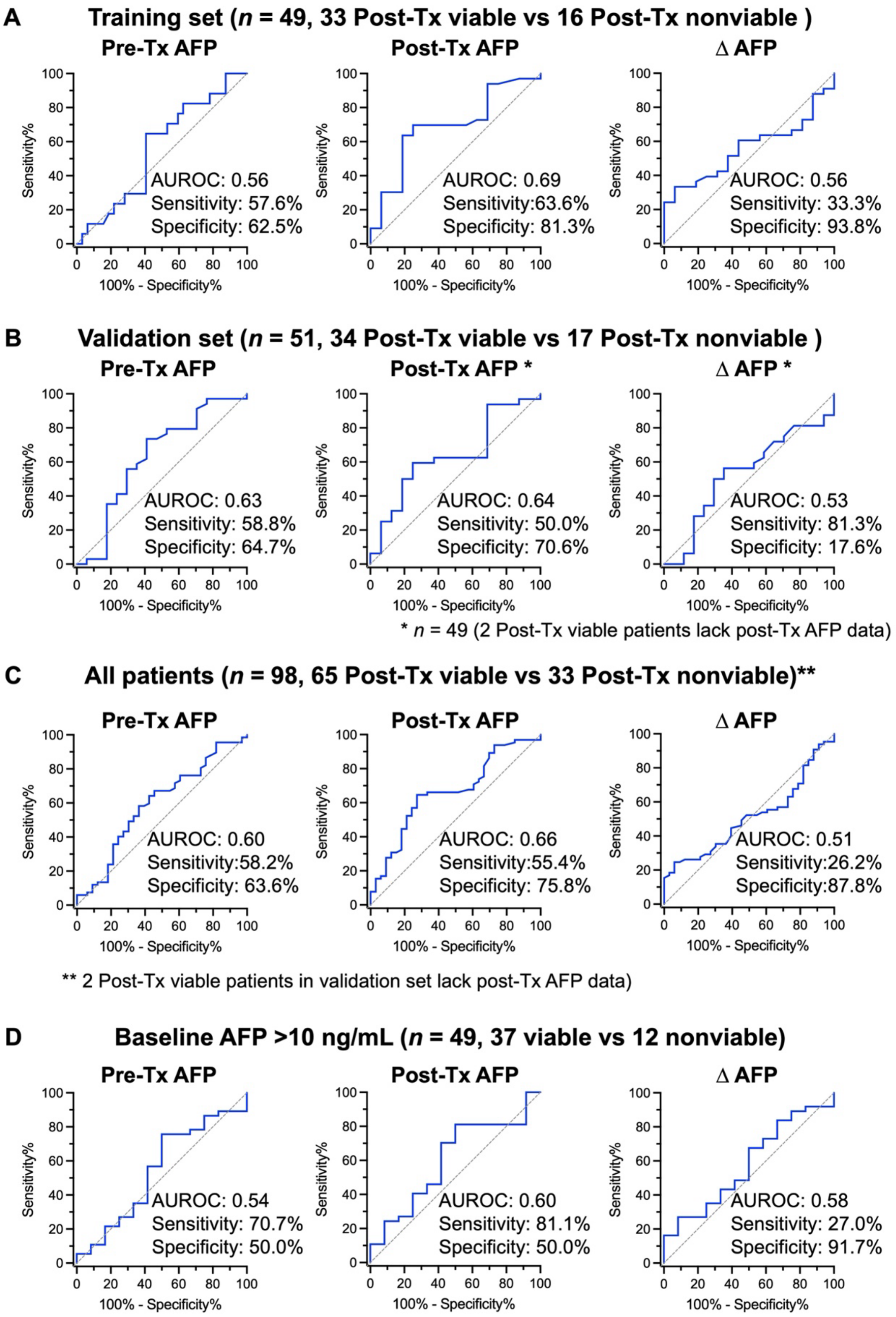


**Fig. S8. ROC curves of serum AFP for differentiating post-Tx viable and nonviable HCC in training set, validation set, all patients, and baseline AFP ≥ 10 ng/mL population.**

ROC curves of serum AFP for differentiating post-Tx viable from nonviable HCC for post-Tx and ΔAFP in **A)** the training set, **B)** the validation set, **C)** all patients, and **D)** baseline AFP ≥ 10 ng/mL population. Post-Tx AFP was obtained a minimum of four weeks following Tx to match sample collection time for the HCC EV Digital Scoring Assay. Δ AFP was calculated by subtracting pre-Tx AFP level from the post-Tx AFP level. Cutoff values in **A-C** were determined by the optimal cutoffs using the training set. Cutoff values in **D** were determined by the optimal cutoffs in baseline AFP ≥ 10 ng/mL population.

AFP, alpha-fetoprotein; Tx, treatment.

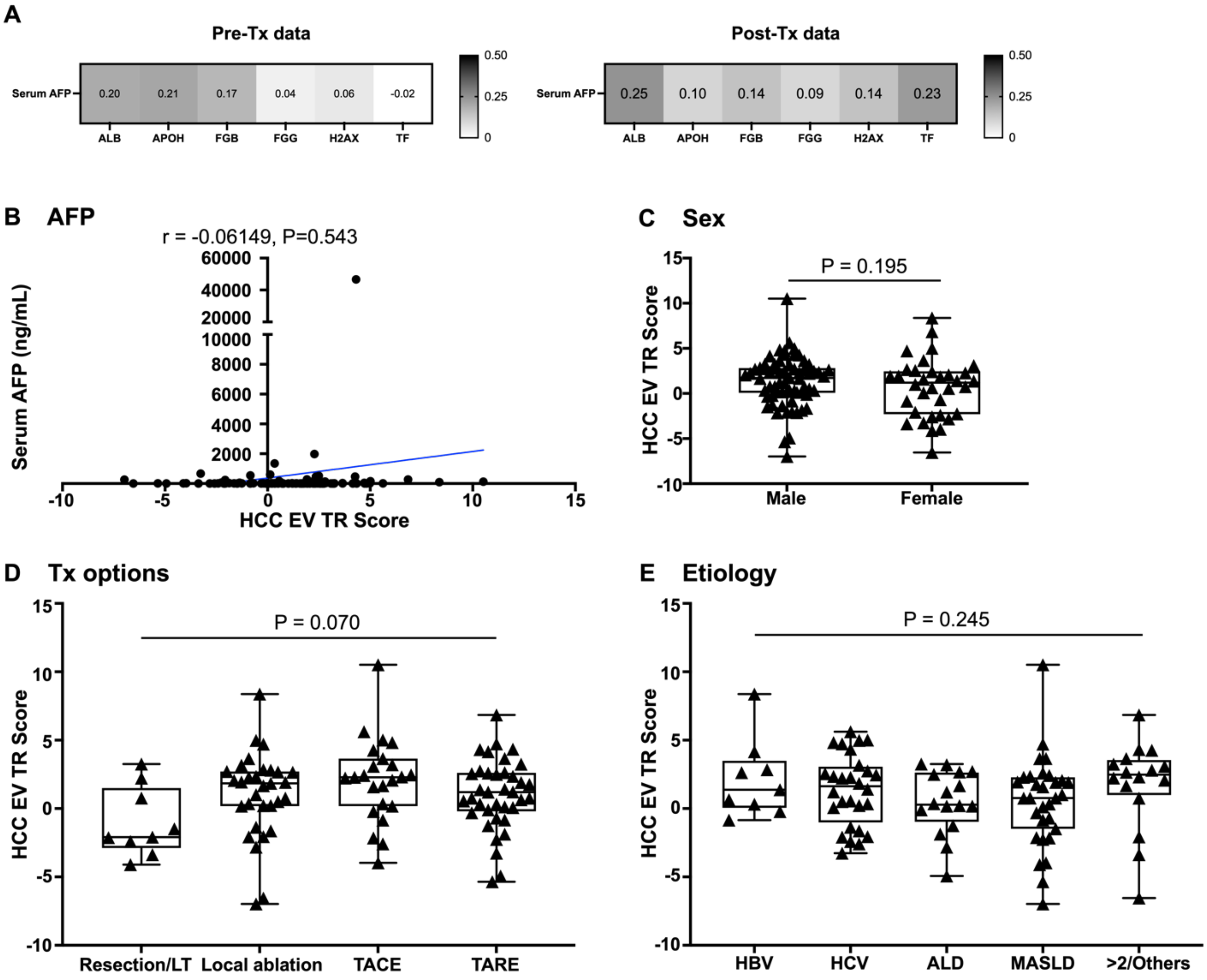


**Fig. S9. Correlation between six HCC EV-specific genes and serum AFP and the association of HCC EV TR Score with serum AFP, sex, Tx options, and liver disease etiology.**
**(A)** Spearman correlation plot between all six HCC EV-specific genes in the HCC EV Digital Scoring Assay and serum AFP from HCC samples before and after treatment. **(B-E)** Associations between HCC EV TR score and **(B)** serum AFP, **(C)** sex, **(D)** Tx options, and **(E)** etiology among HCC patients in the study. Each dot/triangle represents an individual patient.

AFP, alpha-fetoprotein; ALD, alcohol related liver disease; EV, extracellular vesicle; HBV, hepatitis B virus; HCC, hepatocellular carcinoma; HCV, hepatitis C virus; LT, liver transplantation; MASLD, metabolic dysfunction-associated steatotic liver disease; MWA, microwave ablation; RFA, radiofrequency ablation; TACE, transarterial chemoembolization; TARE, transarterial radioembolization; TR, treatment response; Tx, treatment.

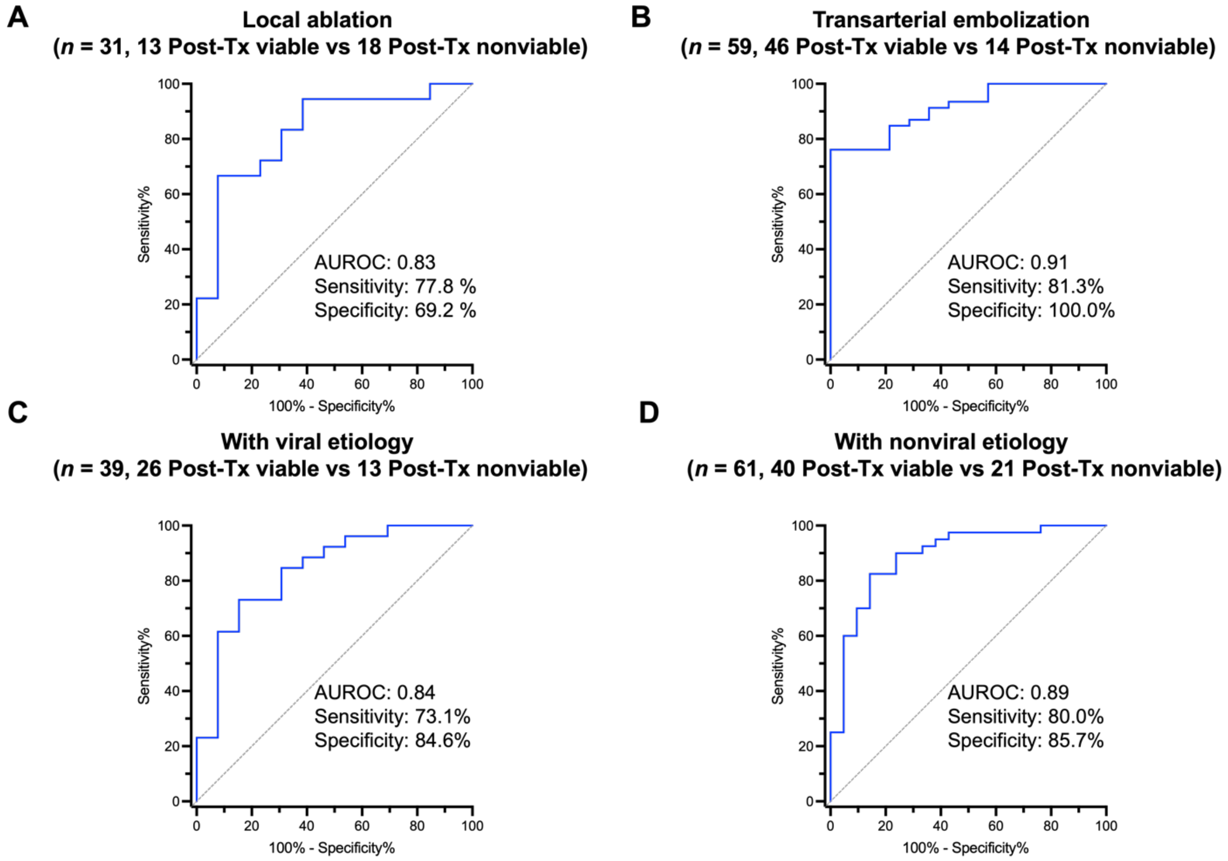


**Fig. S10. The performance of the HCC EV TR Score across subgroups with different treatment modalities and liver disease etiology.**

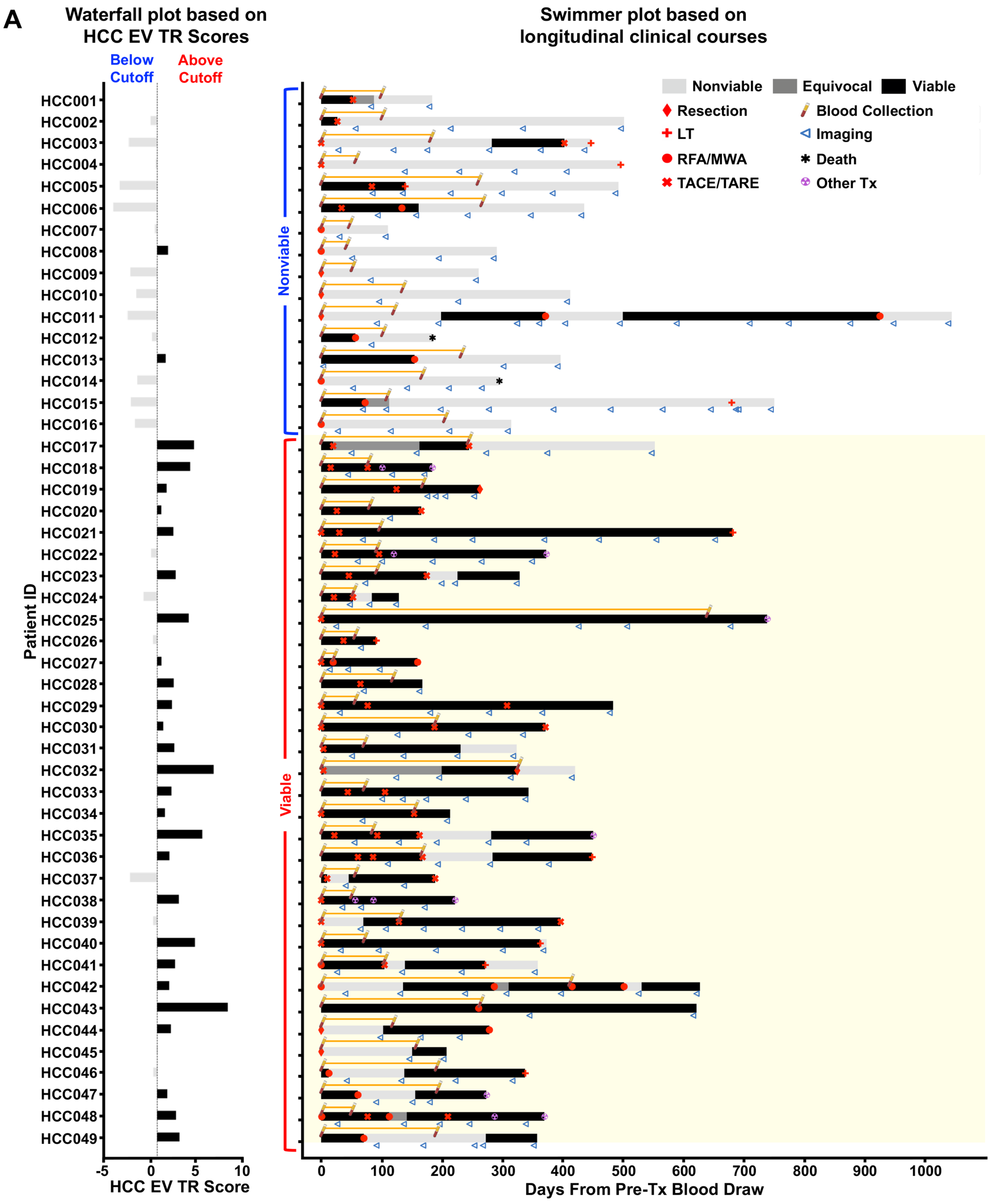

A
Waterfall plot based on HCC EV TR Scores
Below Cutoff
Above Cutoff
Swimmer plot based on longitudinal clinical courses
Nonviable
Equivocal
Viable
Resection
Blood Collection
LT
Imaging
RFA/MWA
Death
TACE/TARE
Other Tx
Patient ID
HCC001
HCC002
HCC003
HCC004
HCC005
HCC006
HCC007
HCC008
HCC009
HCC010
HCC011
HCC012
HCC013
HCC014
HCC015
HCC016
HCC017
HCC018
HCC019
HCC020
HCC021
HCC022
HCC023
HCC024
HCC025
HCC026
HCC027
HCC028
HCC029
HCC030
HCC031
HCC032
HCC033
HCC034
HCC035
HCC036
HCC037
HCC038
HCC039
HCC040
HCC041
HCC042
HCC043
HCC044
HCC045
HCC046
HCC047
HCC048
HCC049
Nonviable
Viable
-5
0
5
10
HCC EV TR Score
0
100
200
300
400
500
600
700
800
900
1000
Days From Pre-Tx Blood Draw

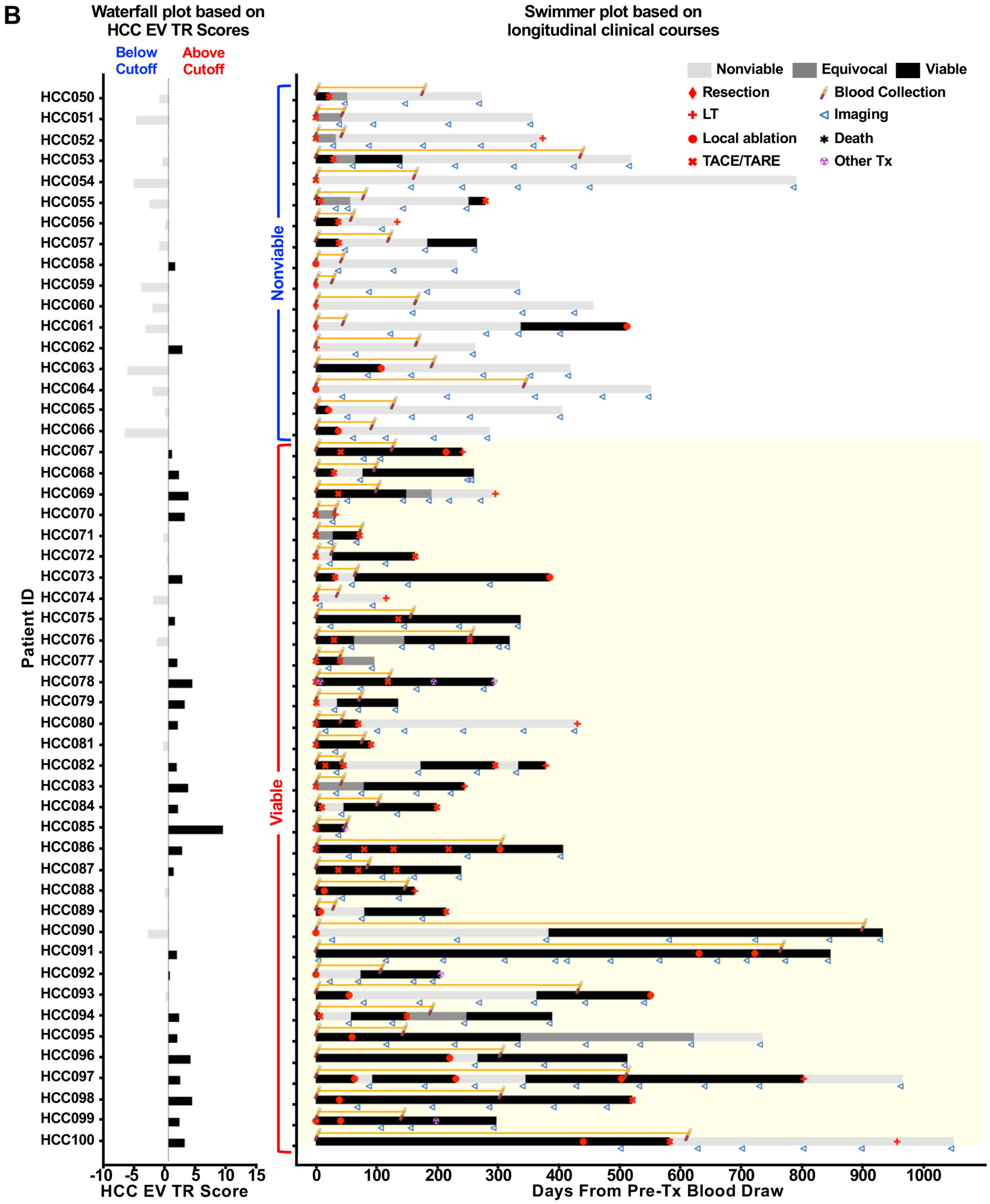


**Fig. S11. Integrated waterfall/swimmer plots summarizing HCC EV TR Scores and clinical course of HCC patients in both training (A) and validation (B) sets.** (Left) The converted waterfall plot presents HCC EV TR Scores, where each horizontal bar represents an individual HCC patient. The color of each bar denotes the patient's HCC EV TR Scores: black (above the optimal cutoff derived from the ROC analysis) or gray (below the cutoff). (Right) The swimmer plot depicts clinical course of HCC patients, including time points of i) pre-Tx and post-Tx blood collection, ii) cross-sectional imaging (CT or MRI), and iii) Tx options. Each horizontal bar represents an individual HCC patient, with color codes denoting the presence (black), equivocal status (medium gray), or absence (light gray) of viable tumor based on cross-sectional imaging assessment per LR-TR algorithm. All events are tracked from pre-Tx blood draw.

EV, extracellular vesicles; HCC, hepatocellular carcinoma; LT, liver transplantation; TACE, transarterial chemoembolization; TARE, transarterial radioembolization; TR, treatment response; Tx, treatment.

## Supplementary Tables

**Table S1. Demographic and clinical characteristics of the 35 early- to intermediate-stage HCC patients (BCLC stage 0-B) in the pilot study.**

| **Characteristic** | ***n* = 35** |
|---|---|
| **Age, median (IQR)** | 65 (49-85) |
| **Male, n (%)** | 24 (68.6%) |
| **Race/ethnicity, n (%)** | |
| Asian | 4 (11.4%) |
| Black | 1 (2.9%) |
| White | 14 (40%) |
| Hispanic | 15 (42.9%) |
| Unknown | 1 (2.9%) |
| **Cirrhosis, n (%)** | 27 (77.14%) |
| **HCC etiology, n (%)** | |
| HBV | 2 (5.7%) |
| HCV | 11 (31.4%) |
| ALD | 9 (25.7%) |
| MASLD | 10 (28.6%) |
| Others | 3 (8.6%) |
| **BCLC stage, n (%)** | |
| Stage 0 | 6 (17.1%) |
| Stage A | 24 (68.6%) |
| Stage B | 5 (14.3%) |
| **Milan criteria, n (%)** | |
| Within Milan criteria | 30 (85.7%) |
| Outside Milan criteria | 5 (14.3%) |
| **AFP, ng/mL, median (IQR)** | 13 (1.2-3656) |

AFP, alpha-fetoprotein; ALD, alcoholic liver disease; BCLC: Barcelona clinic liver cancer; HBV, Hepatitis B virus; HCC, hepatocellular carcinoma; HCV, Hepatitis C virus; IQR, interquartile range; MASLD, metabolic dysfunction-associated steatotic liver disease; Tx, treatment.

**Table S2. Demographic and clinical characteristics of the 35 patients with liver cirrhosis.**

| **Characteristic** | ***n* = 35** |
|---|---|
| **Age, median (IQR)** | 60 (35-82) |
| **Male, n (%)** | 19 (54.3%) |
| **Race, n (%)** | |
| Asian | 2 (5.7%) |
| Black | 0 (0.0%) |
| White | 14 (40.0%) |
| Hispanic | 17 (48.6%) |
| Unknown | 2 (5.7%) |
| **Cirrhosis, n (%)** | 35 (100%) |
| **Etiology, n (%)** | |
| HBV | 1 (2.9%) |
| HCV | 9 (25.7%) |
| ALD | 15 (42.9%) |
| MASLD | 7 (20.0%) |
| Others | 3(8.6%) |
| **AFP, ng/mL, median (IQR)** | 4.0 (1.6-9.9) |

AFP, alpha-fetoprotein; ALD, alcoholic liver disease; BCLC: Barcelona clinic liver cancer; HBV, Hepatitis B virus; HCC, hepatocellular carcinoma; HCV, Hepatitis C virus; IQR, interquartile range; MASLD, metabolic dysfunction-associated steatotic liver disease; Tx, treatment.

**Table S3. Confusion matrix for HCC EV TR score across different Tx options in this study.**

Local ablation ($n$ = 31)

| - | **HCC EV TR Score (cutoff = 0.76)** | | - |
|---|---|---|---|
| **post-LT histology or imaging via LR-TR algorithm** | Predicted Post-Tx viable HCC | Predicted Post-Tx nonviable HCC | - |
| Post-Tx viable HCC | 16 | 3 | Sensitivity = 84.2% |
| Post-Tx nonviable HCC | 3 | 9 | Specificity = 75.0% |
| - | PPV = 84.2% | NPV = 75.0% | Accuracy = 80.6% |

TARE ($n$ = 37)

| - | **HCC EV TR Score (cutoff = 0.76)** | | - |
|---|---|---|---|
| **post-LT histology or imaging via LR-TR algorithm** | Predicted Post-Tx viable HCC | Predicted Post-Tx nonviable HCC | - |
| Post-Tx viable HCC | 19 | 8 | Sensitivity = 70.4% |
| Post-Tx nonviable HCC | 0 | 10 | Specificity = 100.0% |
| - | PPV = 100.0% | NPV = 55.6% | Accuracy = 78.4% |

TACE ($n$ = 23)

| - | **HCC EV TR Score (cutoff = 0.76)** | | - |
|---|---|---|---|
| **post-LT histology or imaging via LR-TR algorithm** | Predicted Post-Tx viable HCC | Predicted Post-Tx nonviable HCC | - |
| Post-Tx viable HCC | 16 | 3 | Sensitivity = 84.2% |
| Post-Tx nonviable HCC | 0 | 4 | Specificity = 100.0% |
| - | PPV = 100.0% | NPV = 57.1% | Accuracy = 87.0% |

Surgery (resection & LT, $n$ = 9)

| - | **HCC EV TR Score (cutoff = 0.76)** | | - |
|---|---|---|---|
| **post-LT histology or imaging via LR-TR algorithm** | Predicted Post-Tx viable HCC | Predicted Post-Tx nonviable HCC | - |
| Post-Tx viable HCC | 2 | 0 | Sensitivity = 100.0% |
| Post-Tx nonviable HCC | 1 | 6 | Specificity = 85.7% |
| -- | PPV = 66.7% | NPV = 100.0% | Accuracy = 88.9% |

EV, extracellular vesicle; HCC, hepatocellular carcinoma; LT, liver transplantation; NPV, negative predictive value; PPV, positive predictive value; TACE, transarterial chemoembolization; TARE, transarterial radioembolization; TR, treatment response; Tx, treatment.

## Table S4. Summary of liquid biopsy detection methods for HCC Tx Response assessment

| Target | Detection method | Study design | | | Performance for distinguishing responder/nonviable from nonresponder/viable | | | Reference |
|---|---|---|---|---|---|---|---|---|
| | | Sample size | Validation set | Stages of enrolled HCC patients | Sensitivity | Specificity | AUROC | |
| **This work (HCC Digital Scoring Assay)** | | | | | | | | |
| HCC EV-specific genes | RT-dPCR | 100 | Yes | early- or intermediate-stage HCC | 76.5% - 81.8% | 87.5% - 88.2% | 0.88 - 0.90 | – |
| **Group 1 – Detection of EV** | | | | | | | | |
| EV Proteins | LC/MS | 45 | No | advanced HCC | N/A | N/A | 0.92-1 | [44] |
| **Group 2 – Detection of ctDNA** | | | | | | | | |
| ctDNA | ddPCR; targeted ultra-deep sequencing | 100 | No | All stage HCC | N/A | N/A | 0.81 | [45] |
| ctDNA | AmpliSeq(Illumina) | 24 | No | unresectable HCC | N/A | N/A | 0.69-0.78 | [46] |
| **Group 3 – Detection of CTC** | | | | | | | | |
| CTC | Single fluorescence image | 48 | No | intermediate-stage HCC | N/A | N/A | 0.70-0.80 | [47] |

**References**


1. Singal AG, Llovet JM, Yarchoan M, Mehta N, Heimbach JK, Dawson LA, Jou JH, Kulik LM, Agopian VG, Marrero JA, et al: **AASLD Practice Guidance on prevention, diagnosis, and treatment of hepatocellular carcinoma.** *Hepatology* 2023, **78:**1922-1965.
2. Marrero JA, Kulik LM, Sirlin CB, Zhu AX, Finn RS, Abecassis MM, Roberts LR, Heimbach JK: **Diagnosis, Staging, and Management of Hepatocellular Carcinoma: 2018 Practice Guidance by the American Association for the Study of Liver Diseases.** *Hepatology* 2018, **68:**723-750.
3. Sun N, Zhang C, Lee YT, Tran BV, Wang J, Kim H, Lee J, Zhang RY, Wang JJ, Hu J, et al: **HCC EV ECG score: An extracellular vesicle-based protein assay for detection of early-stage hepatocellular carcinoma.** *Hepatology* 2023, **77:**774-788.
4. Rinella ME, Lazarus JV, Ratziu V, Francque SM, Sanyal AJ, Kanwal F, Romero D, Abdelmalek MF, Anstee QM, Arab JP, et al: **A multisociety Delphi consensus statement on new fatty liver disease nomenclature.** *Hepatology* 2023, **78:**1966-1986.
5. Barretina J, Caponigro G, Stransky N, Venkatesan K, Margolin AA, Kim S, Wilson CJ, Lehar J, Kryukov GV, Sonkin D, et al: **The Cancer Cell Line Encyclopedia enables predictive modelling of anticancer drug sensitivity.** *Nature* 2012, **483:**603-607.
6. Novershtern N, Subramanian A, Lawton LN, Mak RH, Haining WN, McConkey ME, Habib N, Yosef N, Chang CY, Shay T, et al: **Densely interconnected transcriptional circuits control cell states in human hematopoiesis.** *Cell* 2011, **144:**296-309.
7. Lai H, Li Y, Zhang H, Hu J, Liao J, Su Y, Li Q, Chen B, Li C, Wang Z, et al: **exoRBase 2.0: an atlas of mRNA, lncRNA and circRNA in extracellular vesicles from human biofluids.** *Nucleic Acids Res* 2022, **50:**D118-D128.
8. Bankhead P, Loughrey MB, Fernandez JA, Dombrowski Y, McArt DG, Dunne PD, McQuaid S, Gray RT, Murray LJ, Coleman HG, et al: **QuPath: Open source software for digital pathology image analysis.** *Sci Rep* 2017, **7:**16878.
9. Secci ME, Reed T, Quinlan V, Gilpin NW, Avegno EM: **Quantitative Analysis of Gene Expression in RNAscope-processed Brain Tissue.** *Bio Protoc* 2023, **13:**e4580.
